# Validity and reliability of the pediatric quality of life inventory™ 4.0 generic core scales to measure health-related quality of life in pediatric outpatients after thermal injuries

**DOI:** 10.1186/s41687-026-01247-2

**Published:** 2026-11-11

**Authors:** Clara Herta Spiller, Alexandra Hille, Christina Pählig, Anja Zschieschang, Guido Fitze, Jurek Schultz, Isabel Alkhasli

**Affiliations:** 1 Department of Pediatric Surgery, Carl Gustav Carus Medical Faculty, Technische Universität Dresden, Dresden, Germany; 2 Pediatric and Adolescent Surgery and Urology, Klinikum Chemnitz, Chemnitz, Germany

**Keywords:** Health-related quality of life, PedsQL, Patient-reported outcome measures, Pediatric burns, Psychometric properties, Responsiveness

## Abstract

**Background:**

Health-related quality of life (HRQoL) is a patient-centered outcome in pediatric burn care, yet no burn-specific, validated German-language instrument is available for this population. This study evaluated the feasibility and measurement properties of the German PedsQL™ 4.0 Generic Core Scales (GCS) in pediatric outpatients after thermal injuries.

**Methodology:**

In this retrospective observational study, the authorized German PedsQL™ 4.0 GCS was implemented in a pediatric burn clinic. Data from October 2022 to January 2026 were analyzed across 16 age-, respondent-, and recall-specific forms. Feasibility, internal consistency (model-based omega total), structural validity (ordinal confirmatory factor analysis of parent-proxy reports), floor and ceiling effects, known-groups validity, and responsiveness were assessed. Patients were the unit of analysis; analysis windows (0–30 and ≥ 31 days after the patient-specific index) were distinct from questionnaire recall versions.

**Results:**

Of 1,194 questionnaire opportunities, 951 (79.6%) were completed; language barriers accounted for 42.0% of non-completions. Parent-proxy and child self-report index samples comprised 382 and 134 patients. The parent-proxy Total Score averaged 80.73, with Emotional functioning the lowest (73.61). Of 96 form-by-scale coefficients, 75 were estimated; Emotional functioning point estimates exceeded 0.70 in all forms, although precision was frequently limited, whereas the Total Score was estimable in seven forms (0.814–0.945), and Physical, Social, and Cognitive/School values below 0.70 occurred only in 8–12-year or adolescent forms. No model achieved adequate global fit (correlated four-factor model, *n* = 244: CFI = 0.865; RMSEA = 0.120). Ceiling effects reached 55.2% for Social and 42.0% for Cognitive/School functioning, without relevant floor effects. Known-groups differences were confined to parent-proxy Physical Summary (adjusted *p* = 0.030; δ = −0.238). In 122 paired patients, Total and Physical Summary Scores increased by 4.98 (95% CI 2.80–7.16) and 6.63 (3.45–9.82) points, with no consistent evidence of Social improvement.

**Conclusions:**

The German PedsQL™ 4.0 GCS was feasible for routine use and showed generally adequate internal consistency in several larger form-by-scale analyses. Group-level monitoring requires attention to form, domain, sample size, and structural-fit limitations. Structural validity was only partially supported and examined in parent-proxy reports alone; content validity and invariance were not evaluated. Ceiling effects require cautious interpretation.

**Supplementary Information:**

The online version contains supplementary material available at https://doi.org/10.1186/s41687-026-01247-2.

## Background

Advances in pediatric burn care have shifted attention from injury-centered outcomes toward health-related quality of life (HRQoL) as a central treatment goal. HRQoL monitoring has consequently gained importance in burn research and clinical management [1]. The inclusion of patient-reported outcomes in an internationally developed core outcome set for burn care research further emphasizes the need for validated HRQoL measures for this population [2].

No burn-specific, validated German-language instrument is currently available for assessing HRQoL in pediatric patients with thermal injuries. International studies have used generic measures, including the Pediatric Quality of Life Inventory™ (PedsQL™), and burn-specific measures, such as the Burn Outcomes Questionnaire [3, 4]. However, burn-specific tools are not consistently available across developmental stages and respondent types, whereas the PedsQL™ provides age-adapted parent-proxy and child self-report versions and permits comparison with other pediatric populations [5, 6]. Moreover, concerns reported by children with burns and their caregivers may not be apparent during standard clinical assessment [7]. Approximately half of pediatric burn survivors experience long-term problems involving appearance, emotional well-being, parental concern, or social participation and may require prolonged follow-up and rehabilitation [8, 9].

Effective rehabilitation requires active involvement of patients and families so that children can identify outcomes that matter to them [10]. Patient- and observer-reported outcome measures can capture these perspectives and, in addition to supporting individual care, help characterize disease burden and inform clinical and health-economic decision-making [11, 12].

HRQoL encompasses physical, mental, and social health as well as symptoms, functioning, health perceptions, and individual and environmental factors [13, 14]. Systematic HRQoL assessment can therefore complement traditional outcomes and support patient-centered care [15–18].

The PedsQL™ 4.0 Generic Core Scales (GCS) is widely used to assess pediatric HRQoL. In pediatric surgery, it is among the most frequently reported generic instruments [11]. Although disease-specific PedsQL™ modules are available for several conditions, no pediatric burn-specific module exists. A generic measure was selected because HRQoL after thermal injury is not limited to scar-specific outcomes. Anxiety, appearance-related distress, social participation, and school functioning may remain clinically relevant despite satisfactory wound healing [9].

HRQoL after pediatric burns changes over time through recovery, possible treatment effects, and persistent physical or psychosocial challenges [1, 19, 20]. Instruments used in this setting should therefore not only be feasible in clinical workflows but also demonstrate reliability, structural validity, ability to distinguish clinically relevant groups, and responsiveness to change [21–23]. Because measurement properties and interpretability should be evaluated for the intended population and context of use, evidence from broader pediatric validation samples cannot be assumed to apply to routine pediatric burn aftercare [6, 21, 24–26]. This study evaluated feasibility in routine care, reliability, structural validity, floor and ceiling effects, construct validity using clinically defined diagnostic groups, and longitudinal responsiveness of the German PedsQL™ 4.0 GCS in pediatric outpatients after thermal injuries.

By providing context-specific evidence for this population, the study may support standardized outcome measurement, which improves comparability across studies and clinical settings [2], and integration of patient- and observer-reported outcomes into pediatric burn care. Systematic HRQoL monitoring may identify patients requiring additional support, make changes over time visible during follow-up, and guide targeted referral to physiotherapy, psychological care, or social services [17].

## Methods

### Study design and setting

This retrospective observational study analyzed routine tablet-based assessments collected from October 2022 to January 2026 at a German university pediatric burn clinic following implementation of the authorized German PedsQL™. The study followed the Declaration of Helsinki.

### Participants and data collection

Eligible patients were aged < 18 years and attended with acute burn/corrosion injuries (ICD-10 T20–T32) or burn-related scars (L90.5, L91.0). All eligible routine assessments were considered; no a priori sample-size calculation was performed. Parent-proxy forms covered ages 1 month–18 years, with additional self-reports at ages 8–18 years, completed before consultation. The acute questionnaire (7-day recall) was generally selected for visits within 14 days of the previous visit and the chronic questionnaire (30-day recall) otherwise. At first visits after recent injury or hospital discharge, the acute questionnaire was used. Allocation was pragmatic and not invariably followed; recall version was therefore adjusted for and examined in sensitivity analyses.

### Instrument

The 16 age-, respondent-, and recall-specific PedsQL™ forms comprised 21 Generic Core items in parent-proxy reports for ages 2–4 years and 23 items in parent-proxy forms for ages 5–18 years and child self-report forms for ages 8–18 years. Items cover Physical, Emotional, Social, and Cognitive/School functioning and are rated on a 5-point scale, reverse-scored, and transformed to 0–100, with higher scores indicating better HRQoL [6]. Scale and summary scores were calculated as the mean of available items when ≥ 50% of their constituent items had been answered [6]. The Infant Scales comprised 36 items at ages 1–12 months and 45 at 13–24 months and included Physical Symptoms in the Physical Summary Score [27]. Emotional Functioning item 3 (Sc113; “I feel angry”) was lost during tablet-to-patient-information-system transfer in all chronic child self- report forms (W6/W8) and in some acute child self-report forms (A6/A8) collected before January 2024. W6/W8 reliability analyses therefore used reduced sets of 4 Emotional, 14 Psychosocial, and 22 Total-score items. A6/A8 analyses retained the full item sets and used scale-specific complete cases from January 2024 onward. Scores otherwise followed the ≥ 50%-answered rule; details are provided in Supplementary Methods and Table S3. The instrument is provided by Mapi Research Trust, Lyon, France [28].​​​​

### Statistical analysis

Analyses used Python and R, and measurement properties were evaluated according to the COSMIN framework and ISOQOL minimum standards [21, 24, 25]. ChatGPT/Codex (OpenAI) supported analysis-code development and manuscript preparation; the authors verified the outputs and retain full responsibility for the work.

Detailed protocols, safeguards, and Tables S1–S9 (Additional file 1), as well as executable syntax and README (Additional file 2), are supplied as Supplementary Material.

Feasibility was summarized at the questionnaire-opportunity, questionnaire-record, and patient levels; a questionnaire was counted as completed when participation was documented, irrespective of score calculability. Cross-sectional analyses used the patient as the unit of analysis. Eligible records required a valid patient identifier and assessment date and at least one valid applicable item, after which either the first eligible assessment across forms of the same respondent type (global index) or within each questionnaire form (form-specific index) was selected. Table 1 reports the unit of analysis, selection rule, and denominator for each analysis. All reported scores were derived from or verified against constituent items under the ≥ 50%-answered rule, without imputation.

#### Reliability

Internal consistency was examined for the Total Score, Physical and Psychosocial Summary Scores, and Emotional, Social, and Cognitive/School functioning, separately for each of the 16 forms, using the first eligible assessment per patient and form, scale-specific complete cases and no imputation.

Reliability of each equally weighted observed item score was estimated using model-based omega total, which, unlike coefficient alpha, does not require equal item loadings [29]. Prespecified correlated common-factor models were fitted to Pearson item correlations by unweighted minimum-residual least squares. Sample item standard deviations returned the coefficient to the original observed-score metric, the denominator was the model-implied score variance, and factor correlations represented covariance among the prespecified domains. The complete formula, factor structures and software implementation are provided in Supplementary Methods. Omega total is distinct from omega hierarchical and does not establish a single general HRQoL factor [30].

Coefficients were reported only when the prespecified safeguards were met; otherwise, they were recorded as not estimated under the specified protocol, with reasons classified separately (Supplementary Methods). An unavailable coefficient therefore does not imply low reliability. Percentile 95% confidence intervals were based on 2,000 patient-level bootstrap replications and withheld when the prespecified bootstrap-success criteria were not achieved. Values of ω ≥ 0.70 were treated as a pragmatic group-level benchmark [21] and interpreted together with interval precision, model diagnostics, and structural validity.

#### Structural validity

Structural validity was examined by confirmatory factor analysis (CFA) because the PedsQL™ has a predefined multidimensional structure [6, 25]. CFA was restricted to parent-proxy reports. The first eligible assessment per patient was selected before applying age and completeness restrictions, and acute and chronic versions were pooled because they assess equivalent domains and items. Pooling age groups and recall periods permits evaluation of the common item–factor structure but precludes invariance testing.

Because item responses are ordinal, robust ordinal CFA was performed with lavaan using weighted least squares mean- and variance-adjusted estimation (WLSMV) and theta parameterization [31, 32]. The primary analysis used the 21 items common to parent-proxy versions for ages 2–18 years, including the three kindergarten-related School items of the 2–4-year forms; a sensitivity analysis for ages 5–18 years used all 23 items, with complete cases only. Correlated four-factor, two-factor Physical/Psychosocial, and second-order models were evaluated using scaled χ², CFI, TLI, RMSEA with 90% confidence intervals, and SRMR, with conventional thresholds used descriptively [33, 34]. Robust maximum likelihood and exploratory factor analyses with parallel analysis provided sensitivity checks [35–37]; specifications, parameters and diagnostics are provided in Supplementary Methods and Tables S7–S9.

#### Floor and ceiling effects

Floor and ceiling effects, defined as scores of 0 and 100, were examined for each form using the form-specific index record, with the number of calculable scores per form and scale as denominator (Supplementary Table S4); proportions ≥ 15% were considered relevant [24]. Item missingness and score availability were summarized separately (Supplementary Methods).

#### Longitudinal responsiveness

Responsiveness analyses used all completed parent-proxy records with a valid patient identifier and assessment date. Potential duplicates were grouped by patient, assessment date, and questionnaire form before the analysis windows were assigned; the record with the largest number of valid applicable items (in the event of a tie, the first stable source record) was retained, and no conflicting duplicate groups were observed (Supplementary Methods). Each patient’s earliest retained parent-proxy assessment defined the global index date; assessments 0–30 days after this date formed the acute assessment window and those at ≥ 31 days the follow-up assessment window. These windows indicate time since the patient-specific index assessment and are independent of the PedsQL™ questionnaire recall versions.

For each outcome, paired analyses used the earliest calculable score in each window and included each patient once. Age group was defined from the selected acute-window assessment. Paired t tests, pointwise 95% confidence intervals, Hedges’ g, and standardized response means described follow-up-minus-acute change [22, 23]. Sensitivity analyses retained the same recall version, the same age-specific form, or both (Supplementary Table S5).

Linear mixed-effects models used maximum likelihood, patient-specific random intercepts, conditionally independent homoscedastic residuals, and adjustment for recall version, baseline age group, and sex. Binary-window models used all calculable records from patients represented in both windows; continuous-time models used those from patients with at least two distinct score dates. Wald inference and patient-clustered OLS sensitivity analyses are detailed in Supplementary Methods and Table S6. Holm adjustment [38] covered six outcomes separately for each paired population and model specification, and six age groups within each outcome for age-specific tests; all confidence intervals were pointwise and not adjusted for multiplicity.

#### Construct validity

Construct validity was assessed by known-groups comparisons [21, 24]. Acute burn-related and scar-related diagnoses were defined a priori as clinically distinct diagnostic groups. We expected lower scores in acute assessments, with the largest difference in Physical functioning, because these more often involve wound symptoms and temporary functional limitations [1, 19, 39]. Total and Psychosocial scores were secondary comparisons [1, 8, 19, 20, 40]. Groups were compared using Mann–Whitney U tests with Holm adjustment across the seven estimable outcomes in the original protocol and Cliff’s delta [41]; diagnostic-supergroup analyses and robust regression assessed consistency. Because clinically validated burn depth, total body surface area, detailed anatomical site, treatment intensity and time since injury were unavailable, the comparison contrasts two diagnostic stages rather than severity-matched groups.

## Results

### Sample characteristics

**Fig. 1:**
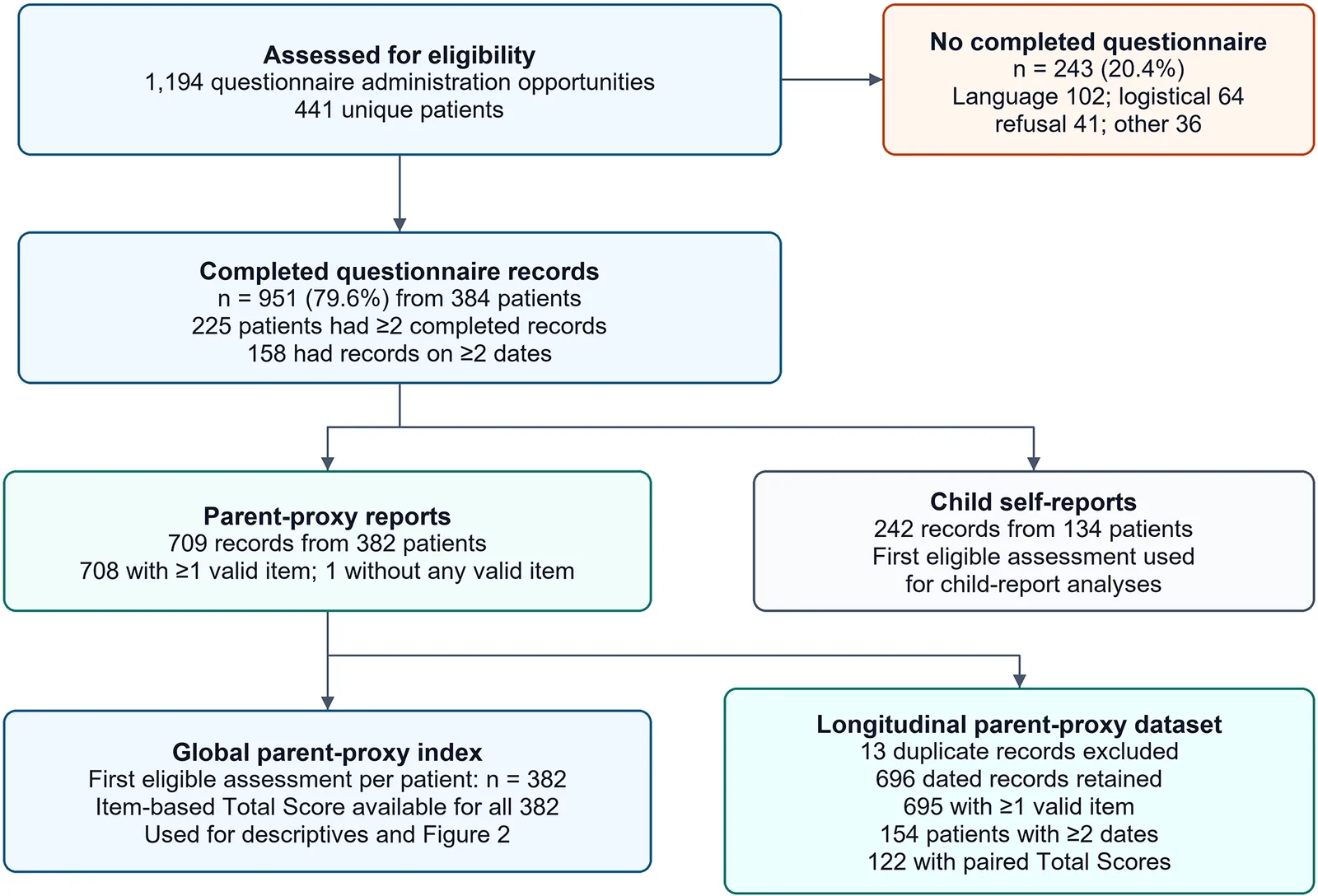
*Flowchart of patient inclusion, exclusion and the final analytical sample*. Overview of screening, exclusions with reasons, and the final dataset used for analysis, including differentiation by respondent type and identification of repeated measurements. Exclusions are grouped into implementation failures (never administered or never returned) and analytical exclusions (one returned parent-proxy record without any valid item, retained in completion counts but not scorable)

Of 1,194 questionnaire administration opportunities involving 441 eligible patients, 951 questionnaires (79.6%) from 384 patients were completed, comprising 709 parent-proxy and 242 child self-report records (Fig. 1). Of the 709 parent-proxy records, 708 contained at least one valid applicable item; one contained none and was retained in questionnaire-flow counts only. The 243 non-completed opportunities reflected implementation rather than psychometric failures, most often language barriers (102, 42.0%; further reasons in Fig. 1).

Parent-proxy respondents were the mother in 504 of 709 records (71.1%), the father in 184 (26.0%), and another respondent in 21 (3.0%). Of the 384 patients, 219 (57.0%) were male and 200 of 382 parent-proxy index patients (52.4%) were younger than 5 years (Table 2). Of the 951 completed assessments, 741 (77.9%) were obtained at regular outpatient consultations, 191 (20.1%) at post-inpatient follow-up visits, and 19 (2.0%) during inpatient or day-clinic stays. At the patient level, trunk injuries were the most frequent diagnostic group (64/384, 16.7%), while 54 patients (14.1%) had a scar-related diagnosis. Body region was available only from the single ICD-10-GM main diagnosis code, which records one predominant region (T20–T25) or none (T29–T30) and cannot represent multiple affected sites, burn extent or clinically validated depth; it was therefore reported descriptively but not used for adjustment. Across both respondent types, 225 patients (58.6%) had at least two completed questionnaire records, 158 of them on at least two distinct dates and 67 on only one date (maximum 16 records per patient).

Across 659 form-specific index questionnaires, 234 of 18,118 intended applicable item responses were missing (1.3%). Of these, 130 (55.6%) concerned the child-report item Sc113: 106 in W6/W8, 23 in A6/A8 before January 2024, and one in a later, extensively incomplete A6 questionnaire. At least one item was missing in 155 questionnaires (23.5%), of which 154 still yielded a Total Score (658/659 calculable, 99.8%); one questionnaire yielded none of the six reported scores. Across 3,954 potential scale and summary scores, 463 were calculable despite missing intended items and 18 were unavailable (0.5%); 12 questionnaires had at least one unavailable score (1.8%).

**Table 1:** Analysis Samples, Units of Analysis and Denominators

Analysis	Unit of analysis	Sampling rule	Denominator
Feasibility and completion (Fig. 1)	Questionnaire administration opportunity	All opportunities screened during the study period; completion required documented participation	1,194 opportunities from 441 patients; 951 completed by 384 patients (709 parent-proxy, 242 child self-report). Of the parent-proxy records, 708 contained ≥ 1 valid item and 1 contained none.
Descriptive statistics (Table 2, Table S1)	Patient	First eligible parent-proxy assessment per patient, pooled across forms (global index)	382 patients (Total Score *n* = 382)
Rank-ordered Total Score distribution (Fig. 2)	Patient	Global parent-proxy index with a calculable Total Score; follow-up assessments excluded	382 patients
Internal consistency (Table 3, Table S2)	Patient within questionnaire form	First eligible assessment per patient within each of the 16 forms; scale-specific complete cases	659 form-specific index records from 384 patients; form-specific index *n* = 13–72 and scale-specific complete-case *n* = 12–70
Floor and ceiling effects (Fig. 3, Table S4)	Patient within questionnaire form	As for internal consistency; scores calculated under the ≥ 50%-answered rule	659 form-specific index records from 384 patients; score-specific denominators reflect item availability and calculability
Structural validity, primary 21-item CFA (Table 4)	Patient	Global parent-proxy index; forms for ages 2–18 years; complete responses to all 21 shared items	244 of 257 age-eligible patients
Structural validity, 23-item sensitivity CFA (Table 4)	Patient	Global parent-proxy index; forms for ages 5–18 years; complete responses to all 23 items	179 of 182 age-eligible patients
Known-groups comparisons (Table 6; Fig. 7)	Patient within respondent stratum	First eligible assessment per patient within each respondent stratum	382 parent-proxy patients (Total Score *n* = 382) and 134 child self-report patients (any calculable reported score *n* = 133)
Longitudinal responsiveness (Table 5; Figs. 4, 5 and 6)	Record for longitudinal models; patient pair for responsiveness	All eligible dated parent-proxy records after same-patient/date/form duplicate resolution; windows defined relative to the patient-specific index assessment	696 retained records from 382 patients (695 with ≥ 1 valid item); 154 patients with ≥ 2 distinct assessment dates; 122 with paired Total Scores in both windows

Note. Denominators differ because each analysis applies its own eligibility, item-availability, and completeness criteria. For the longitudinal flow, 13 additional records from three same-patient/date/form duplicate groups were removed after one record per group was retained; all duplicate groups had identical item responses. The single completed parent-proxy record without a valid applicable item remains in record-flow counts (696 records) but not in score-contributing counts (695 records). No cross-sectional analysis combined repeated questionnaires from the same patient. CFA = confirmatory factor analysis

### Descriptive statistics

Table 2 presents PedsQL™ scores for the global parent-proxy index sample (*N* = 382). The mean Total Score was 80.73 (SD 12.86); Emotional functioning had the lowest mean (73.61) and Social functioning the highest (87.83).

**Table 2:** PedsQL™ scores by age group and questionnaire recall form: global parent-proxy index sample

Questionnaire form	Index *N*	Total	Physical Summary	Psychosocial Summary	Emotional	Social	Cognitive/School
**Acute questionnaire (7-day recall)**							
1–12 months	30	82.74 (11.30)	86.10 (11.17)	80.09 (13.72)	76.64 (16.57)	87.93 (20.18)	82.54 (18.25)
13–24 months	67	80.81 (10.49)	86.53 (8.42)	76.54 (13.40)	73.16 (13.59)	88.38 (12.38)	74.82 (21.49)
2–4 years	45	82.98 (14.58)	84.15 (18.85)	82.31 (13.89)	74.50 (16.34)	91.08 (16.54)	81.69 (19.70)
5–7 years	25	76.26 (18.97)	71.12 (29.39)	79.00 (16.09)	78.00 (16.01)	85.60 (20.33)	73.40 (18.36)
8–12 years	37	75.37 (16.23)	73.14 (28.24)	76.62 (14.21)	71.35 (17.31)	84.19 (14.55)	75.69 (17.33)
13–18 years	17	80.60 (12.99)	80.70 (22.21)	80.53 (12.75)	78.97 (17.61)	92.65 (9.37)	70.00 (16.86)
**Chronic questionnaire (30-day recall)**							
1–12 months	8	82.12 (9.85)	86.13 (10.31)	78.91 (12.56)	73.96 (14.48)	90.62 (10.02)	82.03 (15.10)
13–24 months	20	76.53 (8.14)	82.17 (8.25)	72.17 (10.69)	67.29 (15.41)	89.44 (10.25)	69.30 (18.58)
2–4 years	30	87.74 (6.45)	91.25 (8.89)	85.58 (8.46)	76.00 (14.59)	93.33 (7.35)	88.61 (10.83)
5–7 years	31	79.14 (13.73)	84.88 (17.14)	76.08 (13.84)	67.42 (15.54)	84.84 (14.35)	75.97 (18.46)
8–12 years	45	81.06 (12.51)	87.43 (14.80)	77.67 (14.51)	72.89 (17.73)	86.22 (15.89)	73.89 (18.80)
13–18 years	27	82.33 (9.35)	89.35 (6.50)	78.58 (12.70)	75.69 (19.15)	82.96 (15.21)	77.04 (14.23)
**All forms**	**382**	**80.73 (12.86)**	**83.96 (17.55)**	78.62 (13.57)	**73.61 (16.23)**	**87.83 (14.83)**	**77.03 (18.61)**

Note. Values are mean (standard deviation). One global first eligible parent-proxy assessment per patient was used (*N* = 382). Scores were recalculated from items under the ≥ 50%-answered rule. Total, Physical Summary, Psychosocial Summary and Emotional *n* = 382; Social *n* = 381; Cognitive/School *n* = 371. Medians, ranges and cell-specific n are reported in Supplementary Table S1

**Fig. 2:**
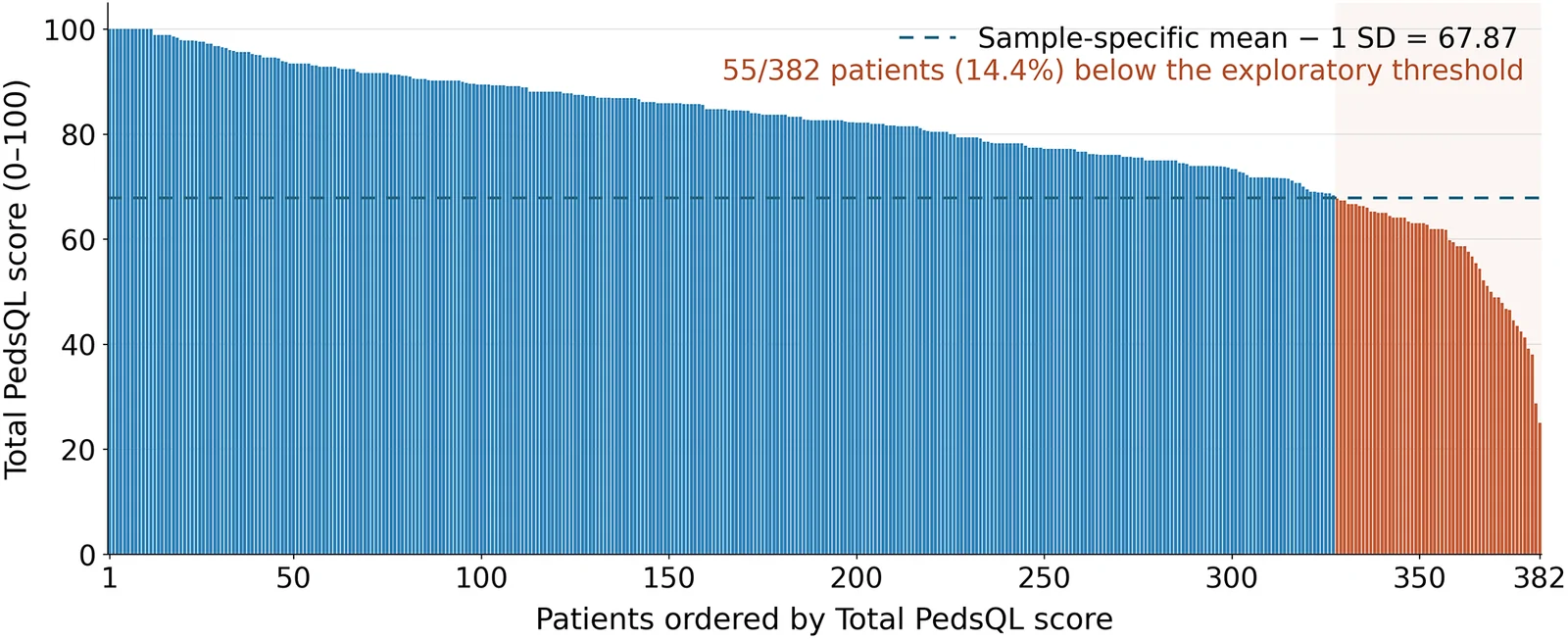
*Rank-ordered distribution of total PedsQL™ scores in the parent-proxy sample*.​ Each vertical bar represents one patient. Patients are ordered by total PedsQL™ score. The dashed horizontal line indicates an exploratory sample-specific threshold of 67.87, calculated from the mean total score minus one standard deviation; it is not a validated clinical cut-off

Fifty-five of the 382 patients (14.4%) scored below the sample-specific threshold of 67.87, defined as 1 SD below the mean (Fig. 2) [5, 42]. Because the threshold was derived from this sample and its particular mix of age-specific forms, it visualizes relative burden and is neither an externally validated clinical cut-off nor an age-independent norm.

### Reliability

Internal consistency was evaluated in 659 form-specific index records from 384 patients, with 12–70 scale-specific complete cases per form (13–70 for the 75 reported coefficients; *n* = 12 occurred only in withheld analyses). Of 96 form-by-scale combinations, 75 yielded omega estimates; 21 were not estimated under the specified protocol because the complete-case sample did not exceed the item count (*n* = 9), an improper/Heywood solution occurred (*n* = 6), or the optimizer reached a parameter boundary (*n* = 6). None was withheld for formal model non-identification.

Among estimable coefficients, 66 of 75 were ≥ 0.70 and 56 were ≥ 0.80 (Table 3). Values below 0.70 occurred in two Physical (0.497 and 0.680), three Social, and four Cognitive/School coefficients, all in the 8–12-year or adolescent forms; all estimable Emotional (0.731–0.936), Psychosocial Summary, and Total Score point estimates exceeded 0.70.

Bootstrap confidence intervals were reportable for 43 estimates; 27 had lower limits ≥ 0.70. Intervals were withheld for 32 estimates because fewer than 500 fits were valid or fewer than 90% of attempted fits succeeded; for example, the acute 2–4-year Total coefficient (0.814) rested on only 350 of 2,000 valid fits and therefore provides limited support. Caution flags applied to 73 of 75 estimates (Supplementary Table S2). Item Sc113 was unobserved in both chronic child self-report forms; their Emotional, Psychosocial Summary, and Total Score coefficients therefore used reduced item sets.

**Table 3:** Model-based internal consistency (Omega Total) of PedsQL™ scales by questionnaire form

Questionnaire form (index N)	Physical ω (*n*)	Emotional ω (*n*)	Social ω (*n*)	Cognitive/School ω (*n*)	Psychosocial Summary ω (*n*)	Total ω (*n*)
**Panel A: parent-proxy reports**						
A1, acute, 1–12 months (30)	0.824* (29)	0.906* (29)	NE (29)	0.816* (29)	0.918* (29)	NE (29)
A2, acute, 13–24 months (72)	0.823* (68)	0.857* (70)	0.739* (70)	0.921* (67)	0.935* (65)	0.945* (63)
A3, acute, 2–4 years (47)	0.888* (46)	0.731* (46)	0.892* (46)	0.742* (37)	0.710* (37)	0.814* (37)
A4, acute, 5–7 years (29)	0.928* (29)	0.763* (29)	0.836* (29)	NE (29)	NE (29)	NE (29)
A5, acute, 8–12 years (41)	0.934* (41)	0.759* (41)	0.624* (41)	0.678* (40)	0.855* (40)	0.914* (40)
A7, acute, 13–18 years (18)	0.914* (18)	0.855* (17)	0.570* (18)	0.516* (18)	0.862* (17)	NE (17)
W1, chronic, 1–12 months (13)	NE (13)	0.828* (13)	NE (13)	NE (13)	NE (13)	NE (13)
W2, chronic, 13–24 months (46)	0.875* (46)	0.890* (46)	0.837* (45)	0.847* (45)	0.913* (45)	NE (45)
W3, chronic, 2–4 years (50)	0.904* (50)	0.865 (50)	0.815* (50)	NE (50)	NE (50)	NE (50)
W4, chronic, 5–7 years (44)	0.881* (44)	0.836* (44)	0.796* (44)	0.809* (44)	0.916* (44)	0.938* (44)
W5, chronic, 8–12 years (69)	0.841* (69)	0.809 (69)	0.760* (69)	0.817* (69)	0.904* (69)	0.920* (69)
W7, chronic, 13–18 years (36)	0.497* (36)	0.863* (35)	0.754* (36)	0.587* (36)	NE (35)	NE (35)
**Panel B: child self-reports**						
A6, acute, 8–12 years (40)	0.916* (39)	0.936* (21)	0.857* (39)	0.881* (39)	NE (21)	NE (21)
A8, acute, 13–18 years (18)	0.926* (18)	0.869* (13)	0.501* (18)	0.600* (17)	NE (12)	NE (12)
W6, chronic, 8–12 years (67)	0.761* (67)	0.818* (67)	0.804* (66)	0.860* (66)	0.927* (65)	0.929* (65)
W8, chronic, 13–18 years (39)	0.680* (38)	0.878* (39)	0.830* (38)	0.849* (39)	0.930* (38)	0.930* (38)

Note. Values are omega total for the equally weighted observed item score; scale-specific complete-case n is in parentheses and index N is the number retained before scale-specific missingness. NE = not estimated under the prespecified protocol because of the pragmatic sample-size rule, an improper/Heywood solution, or an optimizer boundary; no coefficient was withheld for formal model non-identification. NE does not imply zero or low reliability. *Interpret cautiously; reasons and bootstrap confidence intervals are reported in Supplementary Table S2. An unavailable confidence interval indicates that prespecified bootstrap-success criteria were not met. Infant Physical combines Physical functioning and Physical symptoms; Infant Total uses a different item set from the Generic Core forms. W6/W8 Emotional, Psychosocial Summary, and Total coefficients use reduced item sets without Sc113. A6/A8 analyses retained the full item sets; complete cases for these outcomes were available only from January 2024 onward. Acute = 7-day recall; chronic = 30-day recall

### Structural validity

The primary CFA included 244 of 257 age-eligible patients with complete responses to the 21 shared items. No model demonstrated adequate global fit (Table 4); the correlated four-factor and the nested second-order model fit similarly, whereas the two-factor model fit least well.

**Table 4:** Robust ordinal confirmatory factor analysis model fit for independent PedsQL™ parent-proxy index assessments

Analysis	Model	*n*	χ²	df	*p*	CFI	TLI	RMSEA [90% CI]	SRMR
Primary, 21 items	Correlated four-factor	244	819.75	183	< 0.001	0.865	0.845	0.120 [0.111, 0.128]	0.160
Primary, 21 items	Two-factor	244	952.05	188	< 0.001	0.838	0.819	0.129 [0.121, 0.138]	0.169
Primary, 21 items	Second-order	244	805.02	185	< 0.001	0.868	0.851	0.117 [0.109, 0.126]	0.161
Sensitivity, 23 items	Correlated four-factor	179	987.04	224	< 0.001	0.829	0.807	0.138 [0.130, 0.147]	0.187
Sensitivity, 23 items	Two-factor	179	1122.89	229	< 0.001	0.799	0.778	0.148 [0.139, 0.157]	0.190
Sensitivity, 23 items	Second-order	179	971.53	226	< 0.001	0.833	0.813	0.136 [0.127, 0.145]	0.187

Note. Weighted least squares mean- and variance-adjusted (WLSMV) estimation with theta parameterization. Analyses used patients from the global first eligible parent-proxy index sample (*N* = 382) with complete responses to all analyzed items. All six WLSMV models produced the lavaan warning that the variance–covariance matrix of the estimated parameters did not appear to be positive definite; one eigenvalue was at numerical-zero magnitude, whereas expected-information matrices were full rank, indicating numerical near-singularity rather than non-identification or an improper solution (Supplementary Table S8). Coefficient-level standard errors and nominal p-values nevertheless require caution. MLR sensitivity models produced no such warning (Supplementary Table S7). No child self-report form was included, so these results apply to parent-proxy reports only. In the 21-item analysis, the three School items of the 2–4-year forms refer to kindergarten or daycare and partly differ in content from those of the older forms; the 23-item analysis (ages 5–18 years) used content-identical items. CFI = comparative fit index; TLI = Tucker–Lewis index; RMSEA = root mean square error of approximation; SRMR = standardized root mean square residual

Standardized WLSMV item loadings in the 21-item correlated four-factor model ranged from 0.568 to 0.982. All WLSMV models converged and passed admissibility checks, and diagnostics supported numerical near-singularity rather than an improper solution; parameter-level inference nevertheless requires caution (Supplementary Tables S7 and S8).

The 23-item sensitivity analysis (179 of 182 age-eligible patients) reproduced poor global fit, as did MLR analyses (Table 4; Supplementary Table S7), in which Sc214, Sc314, and Sc315 loaded weakly (≤ 0.29). Polychoric parallel analyses retained four and five factors for the 21- and 23-item samples, respectively, whereas Pearson analyses retained four in both. The marginal fifth factor comprised the school-absence items Sc314 and Sc315 and generated an ultra-Heywood warning, whereas Sc214 loaded with the Physical items (Supplementary Table S9).

### Floor and ceiling effects

**Fig. 3:**
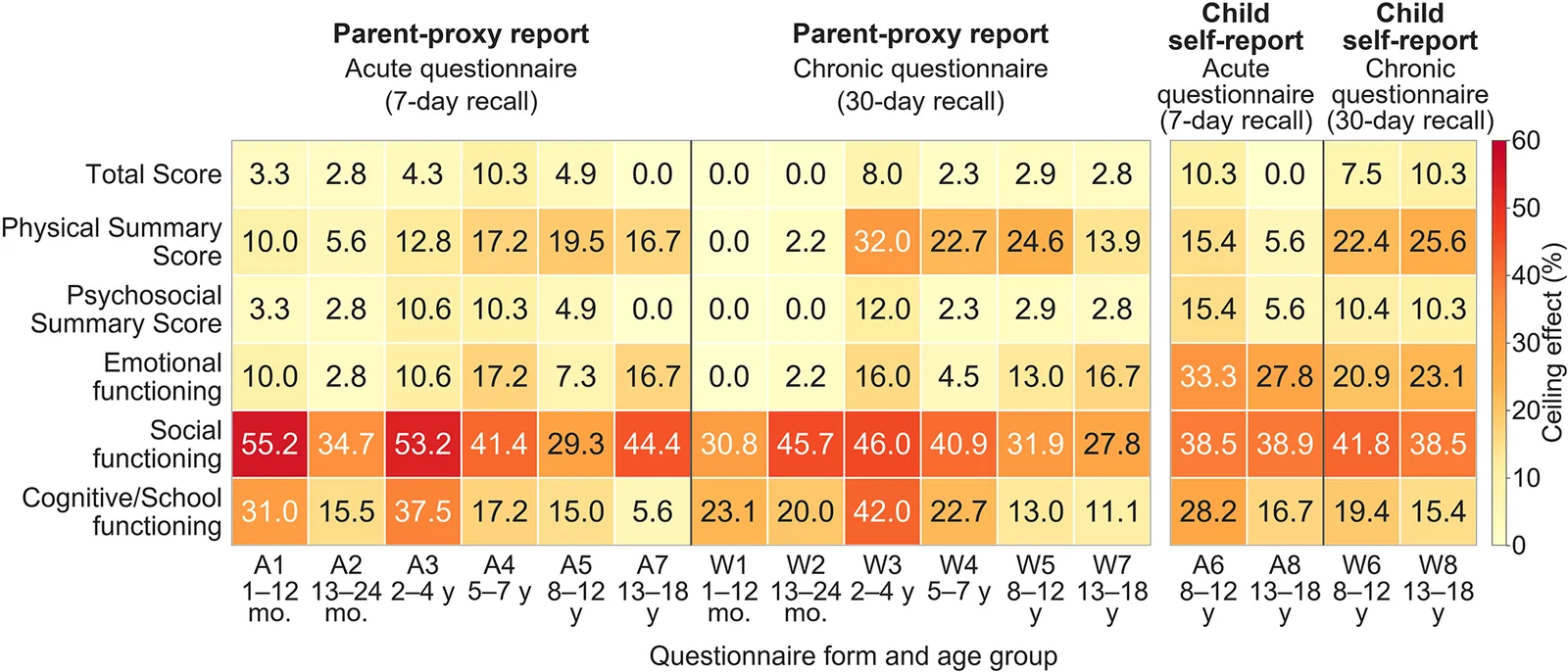
*Ceiling effects across PedsQL domains and questionnaire forms*.​ Values are percentages of calculable scores within each form and scale; numerators and denominators are reported in Table S4. Acute and chronic questionnaires use 7- and 30-day recall periods, respectively. Values ≥ 15% indicate relevant ceiling effects

Among the 659 form-specific index records, no relevant scale-level floor effects occurred (maximum 2.0%). Relevant ceiling effects (≥ 15%) occurred for Social functioning in all 16 forms (27.8%–55.2%) and for Cognitive/School functioning in 13 of 16 forms (5.6%–42.0%), were less consistent for the Physical Summary Score and Emotional functioning (9 and 8 forms), occurred for the Psychosocial Summary Score in only one form (0%–15.4%), and remained below 15% for the Total Score in every form (0%–10.3%; Fig. 3; Supplementary Table S4).

### Longitudinal responsiveness

**Fig. 4:**
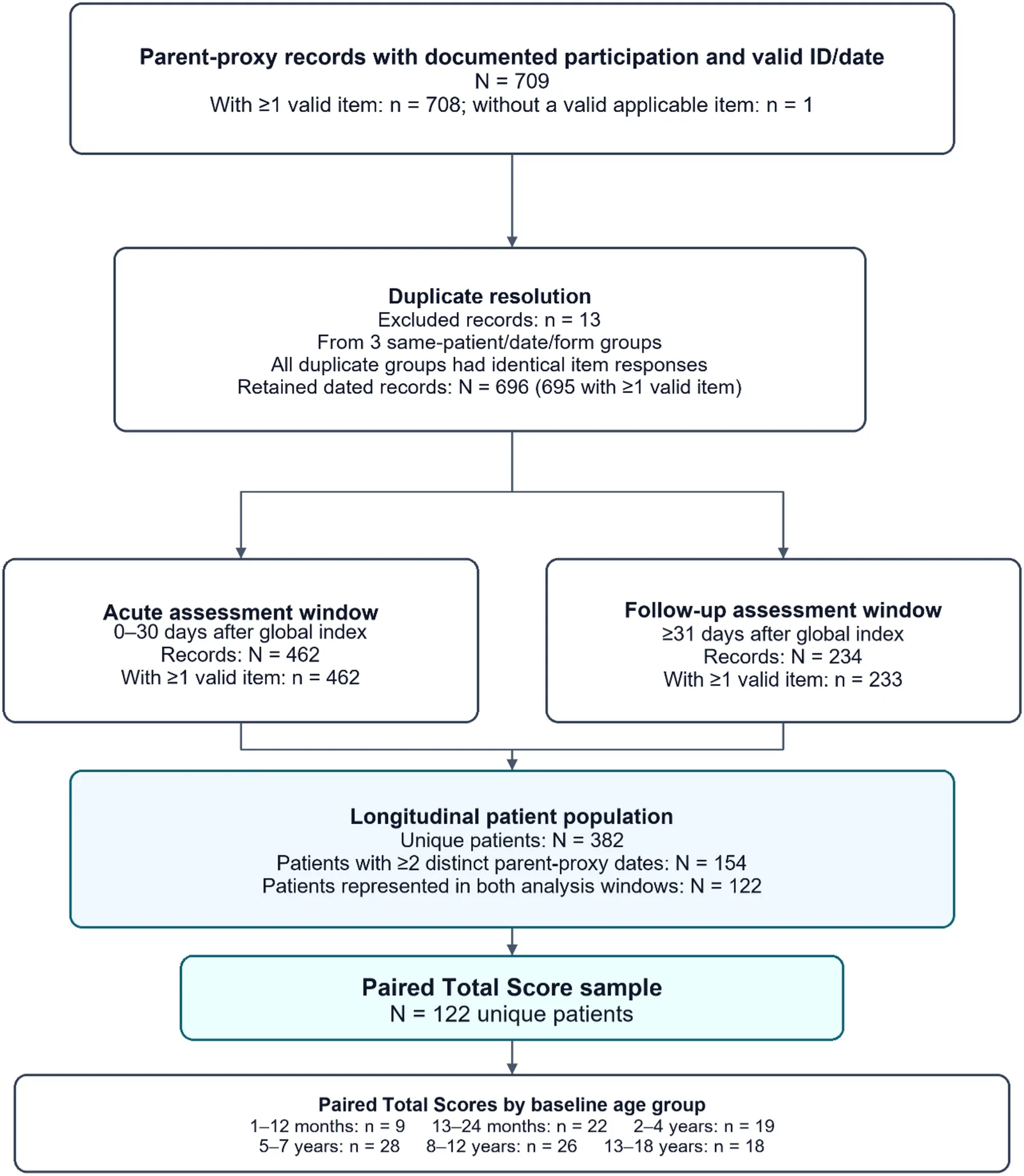
*Flowchart of the longitudinal sample structure. *Overview of questionnaires included in longitudinal analyses, stratified into acute (0–30 days) and follow-up (≥ 31 days) assessments, with identification of patients contributing repeated measurements and distribution across age groups. Windows refer to time since the patient-specific index assessment and not to the PedsQL™ acute and chronic recall versions

After one record per same-patient/date/form duplicate group was retained, 13 additional records from three groups with identical item responses were excluded, leaving 696 of the 709 completed parent-proxy records from 382 patients (462 acute-window and 234 follow-up-window records). One retained follow-up-window record contained no valid applicable item, so the potentially score-contributing pool comprised 695 records (462 and 233). Overall, 154 patients had parent-proxy assessments on at least two distinct dates, and 122 were represented in both analysis windows and contributed paired Total Scores. Age-specific paired samples ranged from 9 to 28 patients (Fig. 4; Table 5).

**Table 5:** Paired changes in PedsQL™ Total scores from the acute assessment window to the follow-up window

Baseline age group	*n*	Mean change [95% CI]	*p*	Hedges’ g	SRM
1–12 months	9	1.3 [− 6.2, 8.7]	1.000	0.12	0.13
13–24 months	22	2.7 [− 1.9, 7.3]	0.904	0.25	0.26
2–4 years	19	0.7 [− 2.6, 3.9]	1.000	0.09	0.10
5–7 years	28	9.8 [4.0, 15.6]	0.011	0.64	0.65
8–12 years	26	7.1 [2.0, 12.1]	0.042	0.54	0.56
13–18 years	18	3.7 [− 2.5, 9.9]	0.904	0.28	0.30

Note. Positive change indicates higher follow-up HRQoL. p values are Holm adjusted across age groups. SRM = standardized response mean. Assessment windows were 0–30 days and ≥ 31 days after the patient-specific index assessment and do not correspond to the PedsQL™ acute and chronic recall versions. Estimates for the 1–12-month group rest on nine paired observations and should be interpreted with caution

The selected assessments were a median of 119 days apart (IQR 57–189; range 33–483). Among 122 paired patients, the mean Total Score increased by 4.98 points (95% CI 2.80–7.16; Holm-adjusted *p* < 0.001; g = 0.41) and Physical Summary by 6.63 (3.45–9.82; *p* < 0.001; g = 0.37). Psychosocial Summary (+ 4.12), Emotional (+ 4.31), and Cognitive/School functioning (+ 5.34; *n* = 121) also increased (all adjusted *p* ≤0.009), whereas Social functioning showed a borderline change (+ 2.35; 0.00–4.69; *p* = 0.050; Supplementary Table S5).

**Fig. 5:**
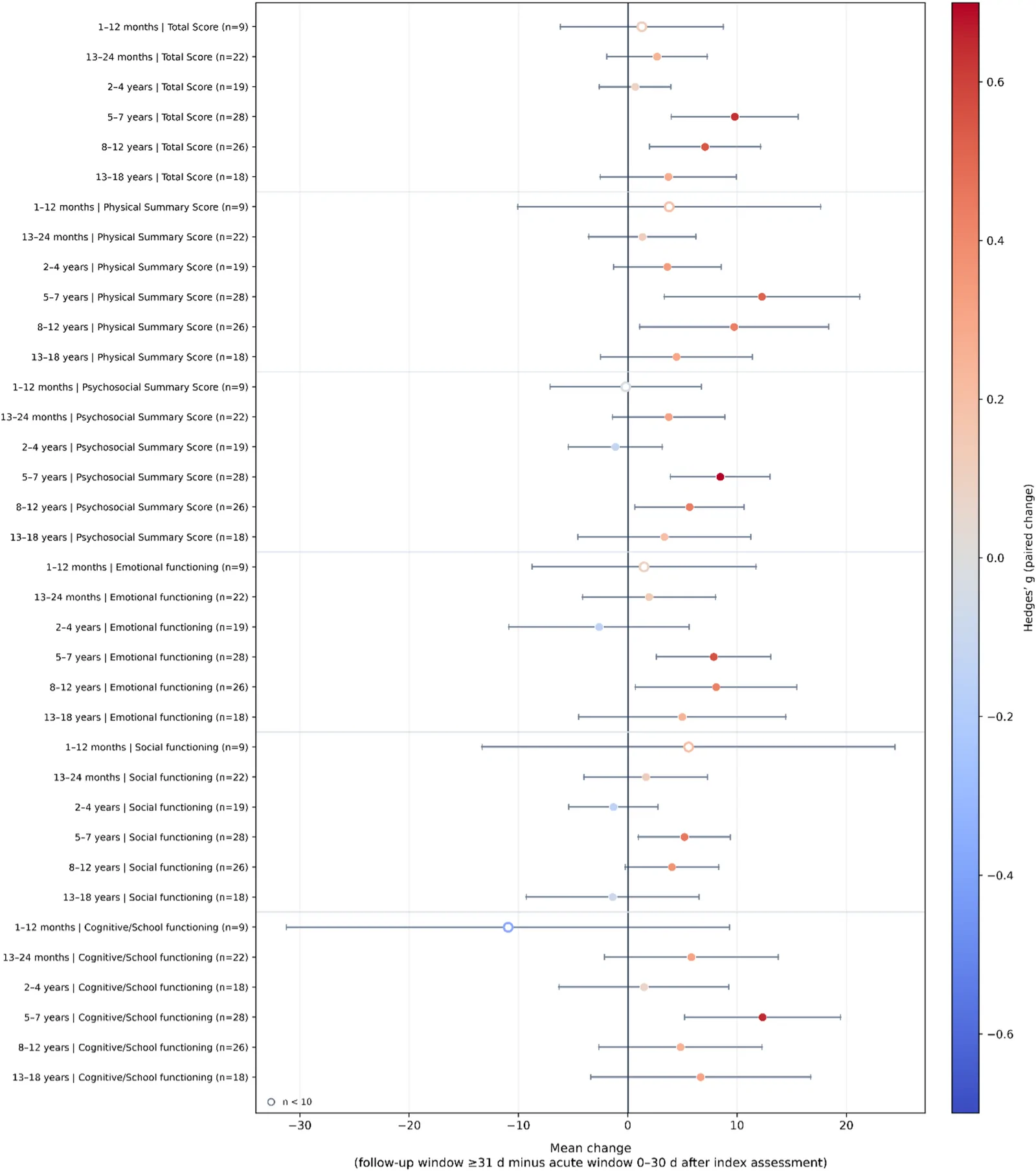
*Paired longitudinal changes in PedsQL™ scores by baseline age group.* Dots show mean paired changes and lines show 95% confidence intervals. Positive values indicate higher follow-up HRQoL. Color represents the paired Hedges’ g for that age group and scale, that is, the mean paired change divided by the standard deviation of the paired differences with small-sample correction. Windows were 0–30 and ≥ 31 days after the patient-specific index assessment, independent of the questionnaire recall version. Open circles mark estimates with *n* < 10, which require caution

**Fig. 6:**
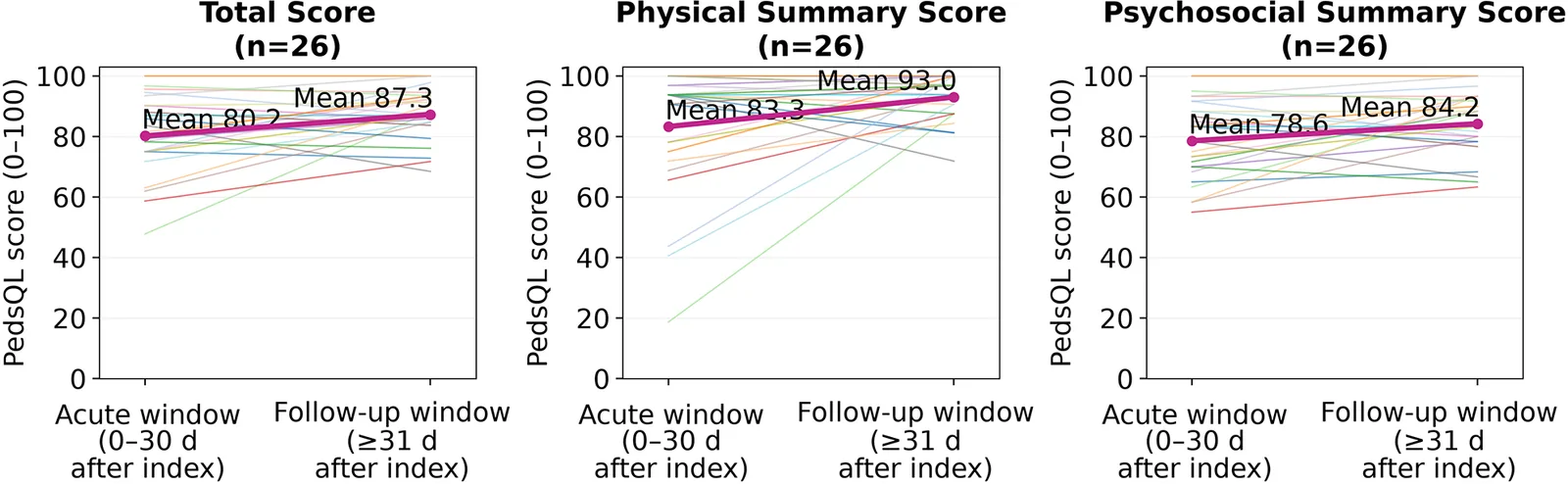
*Individual PedsQL™ trajectories in children aged 8–12 years.* Thin lines show individual paired trajectories and magenta lines show group means (*n* = 26). Higher scores indicate better HRQoL. This age group was selected because it combined one of the largest paired samples with a statistically significant Total-score change. Windows were 0–30 and ≥ 31 days after the patient-specific index assessment, independent of the questionnaire recall version. Changes are observational and do not represent treatment effects

After age-specific Holm correction, Total Scores increased at ages 5–7 years (+ 9.78; *p* = 0.011) and 8–12 years (+ 7.07; *p* = 0.042; Table 5), and at ages 5–7 years Psychosocial Summary (+ 8.45), Emotional (+ 7.86) and Cognitive/School functioning (+ 12.32) also improved (adjusted *p* = 0.004–0.028). No age-specific Physical or Social change remained significant (Physical at 5–7 years: +12.28; *p* = 0.053), and infant estimates rested on nine paired observations (Fig. 5). Figure 6 shows heterogeneous trajectories at ages 8–12 years.

Binary-window mixed models included 121–122 patients and 394–397 observations per outcome. Adjusted follow-up scores were higher for Total (+ 3.78; *p* = 0.008), Physical Summary (+ 4.93; *p* = 0.005), Psychosocial Summary (+ 3.09; *p* = 0.039), Emotional (+ 3.88; *p* = 0.039), and Cognitive/School functioning (+ 3.80; *p* = 0.048), but not Social (+ 0.75, − 1.85 to 3.35; *p* = 0.571). Continuous-time models included 152–154 patients and 462–467 observations; no slope survived Holm correction. All mixed models converged without reportable warnings; cluster-robust OLS results were nonsignificant after adjustment (Supplementary Table S6).

Age-specific forms changed in 24 of 122 Total Score pairs (19.7%; six across Infant age forms, six between Infant and Generic Core forms, twelve between Generic Core age forms), including 14 of 69 same-recall pairs. Restriction to the same age form retained 98 pairs and supported Total, Physical, Psychosocial, Emotional, and Cognitive/School changes. When both recall and age form were unchanged (*n* = 55), only Physical remained significant (+ 4.99, 95% CI 1.58–8.41; adjusted *p* = 0.029); Total change was borderline (+ 3.39, 0.84–5.95; *p* = 0.051; Supplementary Table S5).

### Construct validity

The prespecified hypothesis was supported for parent-proxy Physical Summary Score, which was lower in the acute than in the scar-related group (adjusted *p* = 0.030; Cliff’s δ = −0.238, small), but not for Total or Psychosocial scores, which showed negligible effects (Table 6). Child self-reports showed no significant domain differences, with a small effect for Physical Summary Score (δ = −0.178; adjusted *p* = 0.630).

**Fig. 7:**
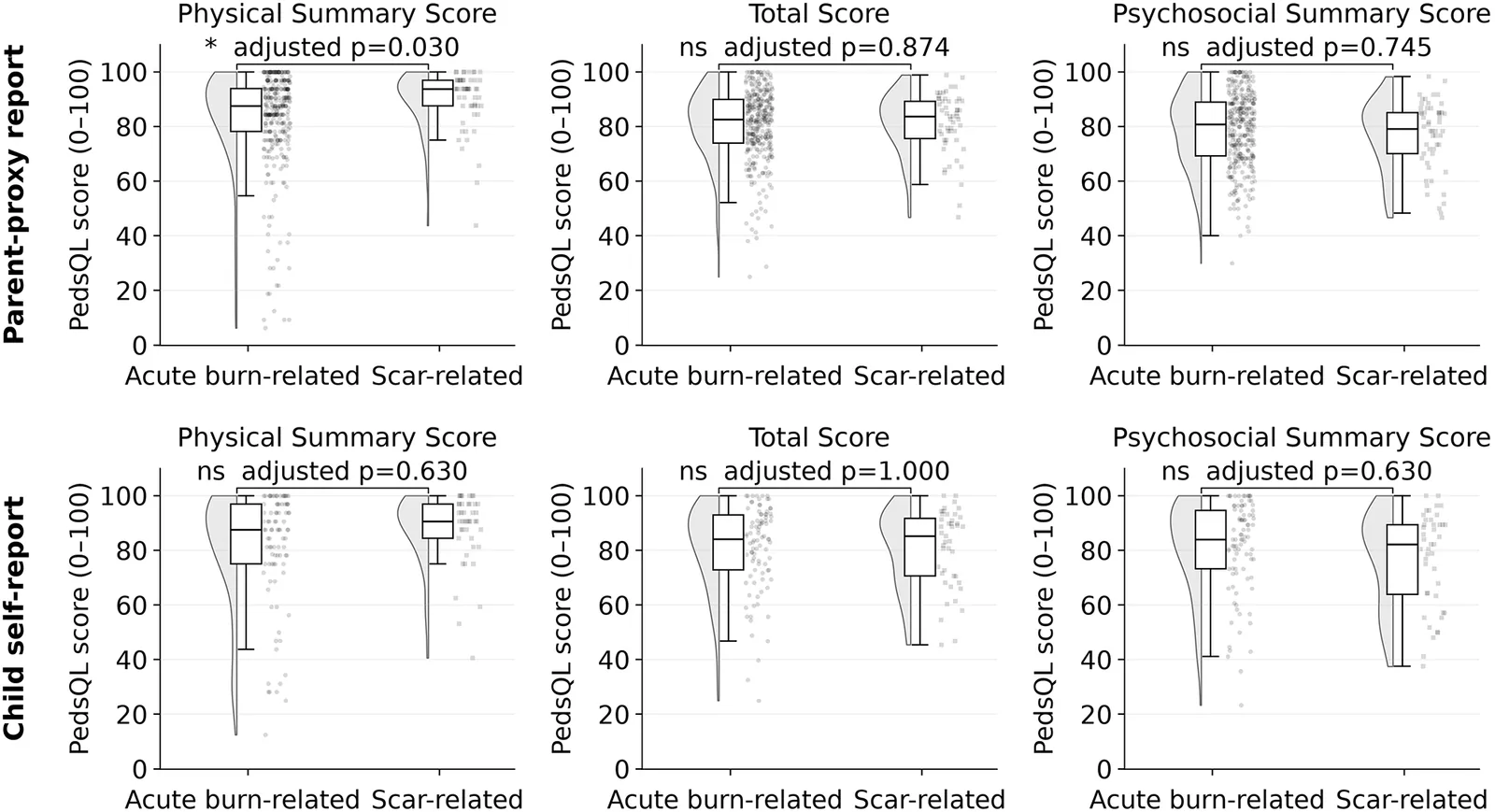
*PedsQL™ scores in acute burn-related and scar-related assessments.* Scores are shown separately for parent-proxy reports and child self-reports. Points represent individual patients (one index assessment per patient and respondent type); violin and boxplots show score distributions, medians, and interquartile ranges. Brackets indicate Holm-adjusted Mann–Whitney U comparisons; “ns” denotes non-significant differences. Groups were not matched or adjusted for burn severity, anatomical site or time since injury

Diagnostic-supergroup analyses identified differences in parent-proxy Physical Summary and the separate Physical Functioning component (both unadjusted *p* = 0.004) but not in Total or Psychosocial scores, and robust regression showed corresponding effects with no consistent child self-report effects (Fig. 7; Supplementary Table S1, Panels C–E). A common-item sensitivity analysis excluding Sc113 from all child self-report forms did not change these findings (all Holm-adjusted *p* ≥ 0.540; Supplementary Table S1, Panel F).

**Table 6:** Known-groups comparisons of PedsQL™ scores between acute burn-related and scar-related diagnosis groups

Outcome	Acute median (*n*)	Scar-related median (*n*)	U	Adjusted *p*	Cliff’s δ	Magnitude	Direction
Parent-proxy report (index *N* = 382)							
Physical Summary Score	87.50 (328)	93.75 (54)	6748.5	0.030	−0.238	small	acute < scar-related
Total Score	82.61 (328)	83.70 (54)	8736.0	0.874	−0.014	negligible	acute < scar-related
Psychosocial Summary Score	80.77 (328)	79.17 (54)	9724.0	0.745	0.098	negligible	acute > scar-related
Child self-report (index *N* = 134)							
Physical Summary Score	87.50 (90)	90.62 (43)	1590.0	0.630	−0.178	small	acute < scar-related
Total Score	84.09 (90)	85.23 (43)	1978.0	1.000	0.022	negligible	acute > scar-related
Psychosocial Summary Score	83.93 (90)	82.14 (43)	2239.5	0.630	0.157	small	acute > scar-related

Note. Medians are followed by the analysis n. Mann–Whitney U tests used one first eligible assessment per patient within each respondent stratum; p values are Holm adjusted across the seven estimable outcomes within each respondent stratum, including the separate Physical Functioning component. Direction follows the sign of Cliff’s δ; magnitude was classified as negligible (|δ| < 0.147), small (< 0.33), medium (< 0.474), or large. Index N is the size of the respondent-specific index sample; the analysis n is smaller where a score was not calculable, and one child self-report index assessment yielded no calculable score (134 patients, 133 scored). Groups were not adjusted for burn depth, total body surface area, anatomical site, treatment intensity, or time since injury

## Discussion

The German PedsQL™ 4.0 GCS could be implemented in routine pediatric burn follow-up and provided internally consistent group-level HRQoL information for most scales. However, only partial structural validity, pronounced ceiling effects in Social and Cognitive/School functioning and coefficients that were not estimable in several small forms indicate that domain scores should not be interpreted in isolation. The age and sex distribution resembled German pediatric burn epidemiology [43].

Unlike parent-proxy reports, child self-reports showed no significant group differences; the child-report Physical Summary effect pointed in the same direction but was smaller and less precisely estimated (δ = −0.178). Because parent and child reports were not compared directly, different significance levels do not establish a difference between informants. The two perspectives may diverge, particularly in psychosocial domains [44, 45], so they should be interpreted as complementary, and including child self-report where developmentally appropriate is supported by prior evidence [46]. Emotional functioning scores were lower than Physical and Social scores, consistent with reports of persistent emotional, appearance-related and participation concerns despite favorable physical recovery [1, 8, 9, 19, 20].

Because the groups were not severity-matched, the parent-proxy Physical Summary difference should not be attributed specifically to acute physical burden; the groups may also differ in severity, treatment history, and referral route. Functional outcomes after pediatric burns are also associated with premorbid functioning and parent-related factors [47].

Internal consistency findings provided qualified support for group-level interpretation of many scales, particularly Emotional and Physical functioning and Total Scores in the larger Generic Core forms. Emotional-functioning point estimates exceeded 0.70 in all 16 forms, and all estimable Psychosocial Summary and Total Score coefficients also exceeded 0.70; however, several confidence intervals were wide or unavailable. The Total Score was not estimated in 9 of 16 forms, mainly under the pragmatic sample-size rule, and 73 of 75 estimates carried at least one caution flag, limiting the support provided by the point estimates alone. Social and Cognitive/School functioning were the least consistent domains, particularly in the 8–12-year and adolescent forms, and should not serve as stand-alone indicators for individual patients.

The CFA did not provide adequate global support for the proposed PedsQL™ structure. No model approached commonly used descriptive criteria; misfit involved Sc214 (unable to do things peers can do) and the school-absence items, which may reflect health-related limitations more than social or school functioning. Shared domain variance therefore does not confirm a single higher-order HRQoL construct, and exploratory analyses did not provide stable support for an alternative structure. The Norwegian validation likewise identified deviations from the expected exploratory structure, particularly for Physical items [46].

Because invariance could not be tested, this pooled parent-proxy evidence does not license comparisons across age groups, recall versions, or respondent types. Reliability and responsiveness may support descriptive group-level monitoring but do not compensate for inadequate structural fit. Larger studies should examine age- and version-specific structures, local item dependencies and invariance, and extend testing to child self-report forms [25, 33, 34]. Because one Emotional Functioning item was systematically unavailable in the chronic child self-report forms, their Emotional, Psychosocial, and Total scores are not fully equivalent to complete-form scores, and their reliability estimates apply only to the reduced item sets; however, common-item sensitivity analyses did not alter the child-report known-groups findings.

Upper-end score compression in Social and Cognitive/School functioning was the main distributional limitation: very high scores neither exclude persistent clinical concerns nor provide a usable screening signal for mild or residual problems, and detectable change may be attenuated [24]. Most parent-proxy scores exceeded published PedsQL™ cut-offs for chronic conditions [42], and the sample-specific threshold identified a lower-scoring subgroup [5]; neither was developed for burn populations, so both remain exploratory.

The PedsQL™ captured average within-patient differences between the analytical windows, but responsiveness varied across age groups, domains, and analyses. Total and Physical Summary changes were supported in primary paired, same-recall, and mixed-model analyses; when both recall and age-specific form were held constant, only Physical remained significant, and cluster-robust models were nonsignificant after adjustment. Form changes affected one fifth of pairs, including transitions between Infant and Generic Core item sets, whose common 0–100 range does not establish equivalence. Social functioning showed no consistent evidence of improvement, alongside pronounced ceiling effects.

The nonsignificant adjusted continuous-time coefficients do not exclude a linear recovery process. Follow-up intervals varied substantially, and observed changes may reflect natural healing, treatment, recall, attendance patterns, and clinical selection; repeatedly assessed children may differ from those assessed once. Covariate adjustment and form-stability analyses address some of these concerns but do not establish longitudinal measurement invariance or treatment effects. Future prospective studies should use standardized injury-referenced intervals, external change anchors, and test–retest data to estimate minimum detectable change.

Clinically, low Physical functioning scores should prompt structured review of pain, range of motion, wound status and scar-related limitations [39], and low Emotional scores should prompt assessment of distress, appearance-related concerns or participation difficulties [1, 9, 19, 20]. Because scores are stored in the medical record, clinicians can review trajectories alongside clinical events, consistent with PROM-based monitoring [15, 18] and outcomes that matter to families [48].

### Limitations

First, this retrospective single-center study was conducted in a German-speaking outpatient clinic, limiting generalizability. Language barriers were a major reason for non-completion and may have introduced selection bias against families with limited German proficiency. Multilingual versions and interpreter-supported administration should therefore be evaluated [49].

Second, several analyses rested on small samples, particularly child self-reports, the infant and adolescent forms and paired longitudinal comparisons; 21 of 96 reliability coefficients were not estimated under the prespecified protocol, and 32 of the 75 estimates lacked a reportable bootstrap confidence interval.

Third, ceiling effects may have attenuated change [24]. Because injury dates and several severity indicators were unavailable, longitudinal windows and diagnostic groups only approximated recovery stage, and severity adjustment was impossible. Content validity, measurement invariance, and test–retest reliability remain to be established, while criterion validity requires a gold standard. Finally, because the instrument is generic, multicenter studies should determine whether burn-specific modules or additional psychosocial screening improve upper-range sensitivity.

## Conclusions

The German PedsQL™ 4.0 GCS was feasible for routine use in pediatric burn follow-up and showed generally adequate internal consistency for group-level interpretation, together with partial change sensitivity that was clearest for Physical functioning. Structural validity was only partially supported and was examined in pooled parent-proxy reports alone; content validity, measurement invariance and test–retest reliability were not evaluated. Pronounced ceiling effects limited discrimination in Social and Cognitive/School functioning. The instrument may be useful for cautious standardized group-level HRQoL monitoring, particularly when Physical and Total scores are interpreted alongside clinical assessment.

## Supplementary Information

Below is the link to the electronic supplementary material.

Supplementary Material 1

Supplementary Material 2

Supplementary Material 3

## Data Availability

The datasets generated and analyzed during the current study are not publicly available because they contain sensitive clinical data from pediatric patients. Data may be available from the corresponding author upon reasonable request and subject to institutional regulations and ethical approval.

## Abbreviations

CFI: Comparative fit index
CFA: Confirmatory factor analysis
COSMIN: Consensus-based Standards for the Selection of Health Measurement Instruments
GCS: Generic Core Scales
HRQoL: Health-related quality of life
MLR: Maximum likelihood estimation with robust standard errors
ω: McDonald’s omega
PROMs: Patient-reported outcome measures
PedsQL™: Pediatric Quality of Life Inventory™
RMSEA: Root mean square error of approximation
SD: Standard Deviation
SRM: Standardized response mean
SRMR: Standardized root mean square residual
TLI: Tucker–Lewis index
WLSMV: Weighted least squares mean- and variance-adjusted estimation
CI: Confidence interval
ICD-10: International Classification of Diseases, Tenth Revision
ISOQOL: International Society for Quality of Life Research
IQR: Interquartile range
OLS: Ordinary least squares
