# Supplementary material for: Validity and reliability of the pediatric quality of life inventory™ 4.0 generic core scales to measure health-related quality of life in pediatric outpatients after thermal injuries
Source: J Patient Rep Outcomes. 2026 Nov 11;10:210. doi: 10.1186/s41687-026-01247-2 (PMC13759929; doi:10.1186/s41687-026-01247-2)
Supplement: Supplementary file 1 [file 41687_2026_1247_MOESM1_ESM.docx]

**Supplementary Methods and Tables**

This supplement provides the detailed analysis protocols, diagnostic output, and supporting Tables S1–S9 for the revised psychometric and longitudinal analyses.

**Supplementary Methods**

**Sample accounting and duplicate-resolution audit**

All sample counts were generated from a single audit of the final data export and were used consistently for Figures 1 and 4 and Table 1. Questionnaire opportunities and completed records were counted independently of score calculability; documented participation therefore defined the feasibility numerator. Completed records were subsequently classified by respondent type, item availability, patient identifier, and assessment date. Cross-sectional index samples required at least one valid applicable item before selection of the first eligible global or form-specific assessment. For the longitudinal parent-proxy dataset, same-patient/date/form groups were examined before window assignment. The record with the greatest number of valid applicable items was retained, with stable source-row order used to resolve ties; when response signatures were identical, this reduced to retaining the first source record. The duplicate audit documented each retained and excluded record, item-response agreement, and the applied rule. The three observed duplicate groups had identical item responses, and no conflicting group occurred. Records without any valid applicable item were retained only in questionnaire-flow counts. Repeated-record counts were defined separately: 225 patients had at least two completed records across either respondent type, including records from the same date; 158 had completed records on at least two distinct dates across respondent types; and 154 had at least two distinct parent-proxy assessment dates after duplicate resolution. Automated checks verified that 709 parent-proxy records equalled 696 retained records plus 13 excluded duplicates, that the 696 retained records comprised 462 acute-window and 234 follow-up-window records, and that excluding the single record without valid item data yielded 695 potentially score-contributing records (462 acute and 233 follow-up).

Global parent-proxy descriptive and known-groups scores were recalculated from the constituent raw items using the form-specific crosswalk and ≥50%-answered rule, without imputation. The same 382 patients, assessment dates and forms were retained. Five exported Total and Psychosocial Summary scores were missing despite sufficient answered items; a sixth record had discrepant Total and Psychosocial scores and an unexported Cognitive/School score. Item-based scoring yielded 382 Total, Physical Summary, Psychosocial Summary and Emotional scores, 381 Social scores and 371 Cognitive/School scores. All six outcomes were independently checked against the record-level scoring audit. Means, sample SDs (ddof = 1), medians and observed ranges were computed before display rounding. Figure 2 uses the sample mean minus one sample SD (67.872829), with 55 of 382 scores strictly below this exploratory threshold. The 659 form-specific index records constitute a different analysis sample; their missingness and score-calculability counts were unchanged.

Known-groups comparisons retained the original diagnostic grouping (328 acute burn-related and 54 scar-related parent-proxy index assessments) and test protocol. Two-sided asymptotic Mann–Whitney tests used tie and continuity corrections; scores were rounded to 10 decimals only to eliminate machine-precision differences between mathematically identical values. Holm correction retained the original family of seven estimable rank tests per respondent stratum: the six reported scores and the separate Physical Functioning component. The Infant Physical Symptoms comparison was not rank-tested because only one scar-related observation was available. HC3-robust OLS sensitivity models estimated the scar-related minus acute mean contrast with normal-reference confidence intervals and retained the original eight-outcome adjustment family. A separate six-score Holm sensitivity yielded the same substantive conclusions. Diagnostic-supergroup Kruskal–Wallis tests retained groups with at least five score observations; their omnibus p values were unadjusted, as in the original protocol. Parent-proxy rank-test, HC3 and diagnostic-supergroup results are provided in Table S1, Panels C–E; the single-observation Physical Symptoms contrast is not reported. Child-report primary results were not changed by this parent-proxy score correction. HC3 uncertainty for Infant Physical Symptoms was withheld because the single scar-related observation had leverage one. The planned eight-test family was retained conservatively by assigning that unavailable test p = 1 only within the multiplicity calculation; no inferential p value or confidence interval was reported for that coefficient.

**Instrument forms, score construction, and unavailable item**

Sixteen age-, respondent-, and recall-specific forms were administered. The Infant Scales comprised 36 items at ages 1–12 months and 45 items at ages 13–24 months. Their Physical Summary Score combines Physical Functioning and Physical Symptoms. The Generic Core Scales comprised 21 items in the parent-proxy form for ages 2–4 years and 23 items in the parent-proxy forms for ages 5–18 years and the administered child self-report forms for ages 8–18 years. The Generic Core Physical Summary Score comprises the eight Physical Functioning items, whereas the Psychosocial Summary Score combines Emotional, Social, and Cognitive/School functioning. The complete form crosswalk is provided in Supplementary Table S3.

Items were reverse-scored and transformed to a 0–100 scale. Each scale or summary score was calculated as the mean of the available constituent items when at least 50% had been answered. The minimum numbers required for the Physical, Psychosocial, and Total scores were 8, 10, and 18 items at ages 1–12 months; 10, 13, and 23 at ages 13–24 months; 4, 7, and 11 at ages 2–4 years; and 4, 8, and 12 at ages 5–18 years, respectively. Clinical and descriptive scores therefore permitted item-level missingness, whereas reliability analyses used scale-specific complete cases.

During data validation, Emotional Functioning item 3 (Sc113; “I feel angry”) was found to be unavailable in all records from the chronic child self-report forms W6 and W8. Investigation showed that the item had not been intentionally omitted or affected by questionnaire routing; its responses had been lost during transfer from the tablet system to the patient information system and could not be recovered from the analytical data. In the acute child self-report forms A6 and A8, the same transfer error affected Sc113 in all records completed before January 2024, whereas all later records contained the item; this concerned 19 of 40 A6 and 5 of 18 A8 form-specific index records. W6 and W8 scores were therefore calculated from the four available Emotional, 14 Psychosocial, and 22 Total-score items, while retaining the original minimum requirements of 3, 8, and 12 answered items. Reliability analyses excluded the entirely unavailable item before complete-case selection; the resulting coefficients consequently estimate reliability of the reduced item sets rather than the complete forms. For A6 and A8, the complete intended item set was retained, so their Emotional, Psychosocial Summary, and Total complete-case samples comprise only records from January 2024 onward.

Common-item sensitivity analysis. The 134 global child self-report index assessments were matched to their item exports, and the original scores and analysis samples were reproduced before rescoring. Eighty-three index assessments used W6 or W8. Sc113 was then excluded from all four child forms to obtain a common 22-item set, which also removes the partial unavailability of Sc113 in earlier A6 and A8 records, retaining the original minimum requirements of 3 Emotional, 8 Psychosocial, and 12 Total items. Two-sided Mann–Whitney tests compared diagnostic groups, with Holm adjustment across the six reported outcomes. A secondary check using the reduced-item ≥50% thresholds (2, 7, and 11 items) yielded the same results. All comparisons retained 90 acute burn-related and 43 scar-related observations; Table S1, Panel F reports the common-item results. These diagnostic groups are distinct from recall versions and longitudinal windows.

**Factor-based reliability analysis**

**Analysis samples and item sets**

Reliability was evaluated separately for each combination of age-specific questionnaire form, respondent type, and recall version. Recall versions were classified as the acute questionnaire with a 7-day recall period and the chronic questionnaire with a 30-day recall period. Eligibility criteria were applied before selecting the chronologically first eligible assessment for each patient within each questionnaire form. The selected index assessment was fixed before scale-specific complete-case filtering; a later assessment was not substituted when the index assessment contained missing items. Consequently, a patient could contribute to different form-specific analyses but only once to a given coefficient. No missing items were imputed.

The prespecified item sets contained 36 items for the 1–12-month Infant Scale, 45 items for the 13–24-month Infant Scale, 21 items for the 2–4-year Generic Core Scale, and 23 items for the Generic Core Scales for ages 5–18 years. Infant and Generic Core coefficients therefore refer to different instruments and are not directly comparable. If an expected item was entirely unobserved within a form, it was omitted from that analysis and the resulting modified item set was documented.

**Common-factor models and reliability estimand**

Five-category item responses were treated as numeric variables, and models were fitted to Pearson item correlations. The target was the reliability of the equally weighted observed PedsQL score; this was not an ordinal latent-response reliability analysis based on polychoric correlations. For complete cases, the equally weighted item sum, item mean, and corresponding 0–100 PedsQL score differ only by a common affine transformation and therefore have identical reliability.

Prespecified correlated common-factor models were estimated by unweighted minimum-residual least squares. The fitting criterion minimized residuals between the observed and model-implied off-diagonal item correlations. Factor variances were fixed to one. Each item loaded only on its assigned domain, cross-loadings and residual correlations were fixed to zero, and correlations among the prespecified domains were freely estimated subject to a positive-definite factor-correlation matrix.

Single-domain scores were represented by one factor. Infant Physical Summary Scores were represented by two correlated factors for Physical functioning and Physical symptoms. Psychosocial Summary Scores were represented by three correlated factors for Emotional, Social, and Cognitive/School functioning. Total Scores were represented by four correlated factors for the Generic Core Scales and five correlated factors for the Infant Scales.

The estimand was omega total for the equally weighted observed item score. Let Λ denote the standardized factor-loading matrix, Φ the factor-correlation matrix, Θ the diagonal matrix of standardized residual variances, D the diagonal matrix containing the sample standard deviation of each item, and 1 a vector of ones. Omega total was calculated as

ωₜ = [1′DΛΦΛ′D1] / [1′D(ΛΦΛ′ + Θ)D1].

The item standard deviations in D restored the original observed-item scaling after fitting the model to standardized item correlations. The denominator therefore represented the model-implied variance of the equally weighted observed item sum rather than its empirical raw-score variance. For multidimensional scores, the off-diagonal elements of Φ incorporated covariance among the prespecified domains into both the common-score variance and total model-implied score variance. A standardized-item coefficient obtained by setting all item standard deviations to one was calculated only as a supplementary diagnostic and was not the coefficient interpreted in the manuscript. Omega total includes all common-factor variance contributing to the observed score and should not be interpreted as omega hierarchical or as evidence for a single general HRQoL factor.

**Estimation safeguards**

Coefficients were calculated only when the scale-specific complete-case sample met the pragmatic requirements of n ≥ 10 and n > p, where p was the number of analysed items. This sample-size rule was a conservative reporting decision and not a formal model-identification criterion. In the implemented model, formal underidentification was recorded when model degrees of freedom were negative or any specified factor contained fewer than three analysed items. Analyses were rejected when an item had zero variance.

A fitted model was accepted only if the optimizer reported a stationary solution, the optimality criterion was ≤ 1 × 10⁻⁴, no free parameter had an absolute estimate ≥ 9.999, all residual variances exceeded 1 × 10⁻⁶, and the minimum eigenvalue of the factor-correlation matrix was at least 1 × 10⁻⁶. Residual variances at or below the threshold were classified as improper or Heywood solutions. Optimizer failure, a parameter-boundary solution, formal underidentification, a near-singular factor-correlation matrix, zero item variance, and failure of the pragmatic sample-size rule were recorded as distinct reasons for non-estimation. Accordingly, an unavailable coefficient was reported as “not estimated under the prespecified protocol” and did not imply low reliability.

Additional caution flags were retained for small samples (n < 50), omission of an entirely unobserved expected item, model RMSR > .08, residual variances < .01, absolute factor correlations > .95, individual negative loadings after orienting each factor to a positive loading sum, confidence-interval widths > .20, or unavailable confidence intervals. These flags informed interpretation but did not replace the prespecified estimation safeguards.

**Bootstrap uncertainty**

Uncertainty was assessed using 2,000 nonparametric patient-level bootstrap replications within each fixed scale-specific complete-case sample. The same item set and factor structure were retained in every replication. Reproducible form- and scale-specific random seeds were generated from the base seed 20260915. Percentile 95% confidence intervals were calculated from the 2.5th and 97.5th percentiles only when at least 500 bootstrap models were valid and at least 90% of all attempted bootstrap replications produced valid solutions. With 2,000 replications, the latter requirement corresponded to at least 1,800 valid fits. If the original model met all safeguards but the bootstrap-success criterion was not reached, the point estimate was retained and its confidence interval was withheld. Bootstrap failure counts and reasons were recorded for each coefficient.

**Software implementation and reproducibility**

Analyses were conducted in Python 3.12.14 using NumPy 2.3.5, pandas 3.0.1, and SciPy 1.18.1. Common-factor models were fitted with scipy.optimize.least_squares, with a maximum of 1,500 function evaluations per fit. The analysis program exported the patient-selection audit, item audit, factor loadings, factor correlations, coefficient estimates, bootstrap diagnostics, reasons for non-estimation, analysis settings, software versions, and file and script hashes. Before extending the analysis to the Psychosocial Summary Score, the extension procedure verified exact agreement with the previously calculated point estimates, sample sizes, item counts, and model RMSR values and subsequently applied the same protocol to the three-factor Psychosocial Summary models.

**Confirmatory and exploratory factor analyses**

**Analysis samples**

Factor analyses used the global first eligible parent-proxy assessment per patient. Of 709 completed parent-proxy records, 708 had a valid patient identifier and assessment date and at least one valid item response; chronological selection with stable form and source-row tie-breakers yielded 382 unique patient index records. The primary CFA included the 21 items shared by the 2–18-year Generic Core parent-proxy forms: 244 of 257 age-eligible patients had complete responses. In the 2–4-year forms, the three Cognitive/School items refer to kindergarten or daycare and partly differ in content from those of the older forms. They were deliberately retained as indicators of the same domain to maximize the sample, because kindergartens in Germany work pedagogically in a similar way to schools. The 23-item sensitivity analysis used content-identical items. A 23-item sensitivity analysis restricted to the 5–18-year forms included 179 of 182 age-eligible patients with complete responses. No item values were imputed.

**Prespecified CFA models and identification**

Three prespecified models were fitted separately to each item set: (1) correlated Physical, Emotional, Social, and Cognitive/School factors; (2) correlated Physical and Psychosocial factors; and (3) four first-order domain factors loading on a second-order HRQoL factor. Each item loaded only on its assigned factor; cross-loadings and correlated item residuals were fixed to zero. Latent-variable scaling used lavaan's std.lv = TRUE: factor variances were fixed to one in the correlated models; in the second-order model, the HRQoL variance and first-order latent disturbance variances were fixed to one. The theta parameterization fixed ordinal item residual variances to one. Item thresholds and the four higher-order domain loadings were freely estimated, as were the relevant latent correlations in the correlated models. The complete executable model syntax is supplied separately in PedsQL_Executable_Syntax.zip.

**Ordinal estimation, fit measures, and sensitivity analysis**

The primary models treated the 0–4 item responses as ordered and were estimated in lavaan 0.7-2 using WLSMV, theta parameterization, listwise-complete samples, and std.lv = TRUE. The extracted fit measures were chisq.scaled, df.scaled, pvalue.scaled, cfi.scaled, tli.scaled, rmsea.scaled, rmsea.ci.lower.scaled, rmsea.ci.upper.scaled, and srmr. RMSEA intervals used rmsea.ci.level = 0.90. Robust maximum-likelihood sensitivity models treated items as continuous and used MLR, a mean structure, full-information maximum likelihood, and the identical complete-case samples. Their extraction used chisq.scaled (chisq.robust was unavailable), df, pvalue.scaled, cfi.robust, tli.robust, rmsea.robust, rmsea.ci.lower.robust, rmsea.ci.upper.robust, srmr, aic, and bic. Standardized item loadings, higher-order domain loadings, and latent correlations are reported in Table S7. Table S7 includes standardized loadings and latent correlations for both estimators and all three model structures in each sample.

**Numerical diagnostics**

lavaan warned that the parameter covariance matrix 'does not appear to be positive definite' and that its near-zero smallest eigenvalue might indicate nonidentification. Diagnostics therefore examined convergence, lavaan's post-estimation check, the maximum absolute gradient, eigenvalues and numerical rank of the parameter variance–covariance and expected-information matrices, observed residual and latent variances, absolute latent correlations, nonfinite standardized estimates, and standardized loadings greater than one. Numerical rank used a tolerance of 10⁻¹⁰ times the largest absolute eigenvalue. As detailed in Table S8, every WLSMV model had one parameter-covariance eigenvalue at numerical-zero magnitude, while the expected-information matrices remained full rank and no inadmissible variance, correlation, or standardized loading was detected. The corresponding MLR matrices were full rank and generated no warning. These checks support numerical near-singularity rather than demonstrated nonidentification or an improper solution, while retaining caution for parameter-level inference.

**Exploratory parallel analysis**

Exploratory factor and parallel analyses used the same 21- and 23-item complete-case samples. Polychoric item-correlation matrices were estimated without a continuity correction and smoothed when non-positive definite. MINRES factor eigenvalues were compared with the 95th percentile from 1,000 independent random-normal datasets, using prespecified seeds 20261013 and 20261014; factors were retained sequentially until the first observed eigenvalue failed to exceed its reference value. Exploratory models used MINRES extraction and oblimin rotation. Pearson-correlation parallel analyses served as a sensitivity check, using seeds 20261113 and 20261114. Analyses used R 4.6.0, psych 2.6.9, and GPArotation 2026.8.2. The polychoric pattern loadings and factor correlations are included in Table S9. These analyses were hypothesis-generating and were not used to modify the prespecified CFA models (Table S9).

**Floor and ceiling effects**

Floor and ceiling effects were calculated from the first eligible assessment per patient within each questionnaire form. Scores were calculated when at least 50% of their constituent items were answered. For each form and scale, floor and ceiling percentages used the number of calculable scores as their denominator, with the corresponding counts reported in Table S4. Missingness was summarized separately as missing responses among intended applicable items, questionnaires with at least one missing item, and unavailable questionnaire-by-score combinations. Systematically unavailable Sc113 responses were distinguished from other item missingness; score calculation despite a missing intended item was counted separately from score unavailability.

**Longitudinal responsiveness and mixed-effects models**

**Longitudinal record selection and analysis windows**

Longitudinal analyses used parent-proxy records only. The 709 completed records were grouped by patient, assessment date, and form. Within each duplicate group, the record with the greatest number of valid applicable items was retained; tied records were resolved by stable source-row order. All three duplicate groups had identical item-response signatures, and 13 additional records were excluded. This produced 696 dated records from 382 patients, including one record without any valid item response; 695 records could potentially contribute a score. The patient-specific index was the earliest retained parent-proxy assessment. Records 0–30 days after this index formed the acute assessment window and records at least 31 days later formed the follow-up assessment window. These windows were independent of the 7-day acute and 30-day chronic questionnaire recall versions.

**Paired responsiveness and form-stability analyses**

For each outcome, the earliest calculable record in each assessment window was selected, with the greater number of answered constituent items and then stable source order used to break same-date ties. Each patient therefore contributed at most one pair per outcome. Change was defined as follow-up minus acute-window score. Mean change, its pointwise 95% t confidence interval, a two-sided paired t test, paired Hedges g, and the standardized response mean were calculated. Assessment intervals were summarized by median, interquartile range, and range. Sensitivity analyses restricted pairs to the same questionnaire recall version, the same age-specific form, or both. Form transitions were audited separately across Infant Scale boundaries, between Infant and Generic Core forms, and between Generic Core age forms (Table S5).

**Mixed-effects models**

Linear mixed-effects models were estimated separately for the six reported outcomes by maximum likelihood with a patient-specific random intercept and conditionally independent homoscedastic residuals. Models adjusted for questionnaire recall version, baseline age group, and sex when estimable. The binary-window model used all calculable repeated observations from patients represented in both windows and estimated the adjusted follow-up-versus-acute-window difference. The continuous-time model used all calculable observations from patients with at least two distinct score dates and estimated adjusted change per 30 days. Optimization proceeded through L-BFGS, BFGS, and Powell methods until a converged solution without a critical singularity, boundary, or convergence warning was obtained. Model-based Wald standard errors, pointwise 95% confidence intervals, and p values were reported. OLS sensitivity analyses used the same samples and fixed effects, with patient-clustered sandwich standard errors, the statsmodels default finite-sample correction, and pointwise normal-Wald inference. Patient and observation counts, estimates, random-intercept and residual variances, and adjusted p values are reported in Table S6, including the clustered-OLS estimates in Panels C and D.

**Multiplicity**

Holm adjustment was applied to prespecified families while all confidence intervals remained pointwise. The pooled paired tests comprised the six outcomes; each paired sensitivity population constituted a separate six-outcome family. Age-specific paired tests were adjusted across the six baseline age groups separately within each outcome. Mixed-model and cluster-robust OLS p values were adjusted across the six outcomes separately for each time specification and analysis method.

**Supplementary Tables**

**Supplementary Table S1**

*PedsQL™ Score Distributions and Known Groups Sensitivity Analyses*

Panel A. Acute questionnaire (7-day recall)

| **Scale** | **Statistic** | **1–12 mo** | **13–24 mo** | **2–4 y** | **5–7 y** | **8–12 y** | **13–18 y** | **All forms** |
| --- | --- | --- | --- | --- | --- | --- | --- | --- |
| Index N |  | 30 | 67 | 45 | 25 | 37 | 17 | 382 |
| Total | n | 30 | 67 | 45 | 25 | 37 | 17 | 382 |
|  | Mean (SD) | 82.74 (11.30) | 80.81 (10.49) | 82.98 (14.58) | 76.26 (18.97) | 75.37 (16.23) | 80.60 (12.99) | 80.73 (12.86) |
|  | Median | 84.38 | 82.22 | 85.71 | 82.61 | 78.26 | 82.61 | 82.61 |
|  | Min–Max | 46.53–100.00 | 56.67–100.00 | 25.00–100.00 | 39.13–100.00 | 28.75–100.00 | 57.61–97.83 | 25.00–100.00 |
| Physical Summary | n | 30 | 67 | 45 | 25 | 37 | 17 | 382 |
|  | Mean (SD) | 86.10 (11.17) | 86.53 (8.42) | 84.15 (18.85) | 71.12 (29.39) | 73.14 (28.24) | 80.70 (22.21) | 83.96 (17.55) |
|  | Median | 88.28 | 88.16 | 90.62 | 81.25 | 78.12 | 90.62 | 88.16 |
|  | Min–Max | 54.69–100.00 | 64.47–100.00 | 18.75–100.00 | 9.38–100.00 | 6.25–100.00 | 28.12–100.00 | 6.25–100.00 |
| Psychosocial Summary | n | 30 | 67 | 45 | 25 | 37 | 17 | 382 |
|  | Mean (SD) | 80.09 (13.72) | 76.54 (13.40) | 82.31 (13.89) | 79.00 (16.09) | 76.62 (14.21) | 80.53 (12.75) | 78.62 (13.57) |
|  | Median | 83.30 | 79.81 | 84.62 | 80.00 | 75.00 | 85.00 | 80.77 |
|  | Min–Max | 40.00–100.00 | 50.96–100.00 | 30.00–100.00 | 46.67–100.00 | 41.67–100.00 | 55.00–98.33 | 30.00–100.00 |
| Emotional | n | 30 | 67 | 45 | 25 | 37 | 17 | 382 |
|  | Mean (SD) | 76.64 (16.57) | 73.16 (13.59) | 74.50 (16.34) | 78.00 (16.01) | 71.35 (17.31) | 78.97 (17.61) | 73.61 (16.23) |
|  | Median | 79.17 | 72.92 | 75.00 | 80.00 | 75.00 | 85.00 | 75.00 |
|  | Min–Max | 35.42–100.00 | 45.45–100.00 | 35.00–100.00 | 55.00–100.00 | 40.00–100.00 | 40.00–100.00 | 15.00–100.00 |
| Social | n | 29 | 67 | 45 | 25 | 37 | 17 | 381 |
|  | Mean (SD) | 87.93 (20.18) | 88.38 (12.38) | 91.08 (16.54) | 85.60 (20.33) | 84.19 (14.55) | 92.65 (9.37) | 87.83 (14.83) |
|  | Median | 100.00 | 95.00 | 100.00 | 95.00 | 85.00 | 95.00 | 93.75 |
|  | Min–Max | 25.00–100.00 | 60.00–100.00 | 15.00–100.00 | 35.00–100.00 | 50.00–100.00 | 70.00–100.00 | 15.00–100.00 |
| Cognitive/School | n | 29 | 66 | 38 | 25 | 36 | 17 | 371 |
|  | Mean (SD) | 82.54 (18.25) | 74.82 (21.49) | 81.69 (19.70) | 73.40 (18.36) | 75.69 (17.33) | 70.00 (16.86) | 77.03 (18.61) |
|  | Median | 87.50 | 79.56 | 83.33 | 75.00 | 75.00 | 70.00 | 80.00 |
|  | Min–Max | 31.25–100.00 | 25.00–100.00 | 33.33–100.00 | 35.00–100.00 | 40.00–100.00 | 40.00–100.00 | 25.00–100.00 |

Supplementary Table S1 (continued)

Panel B. Chronic questionnaire (30-day recall)

| **Scale** | **Statistic** | **1–12 mo** | **13–24 mo** | **2–4 y** | **5–7 y** | **8–12 y** | **13–18 y** | **All forms** |
| --- | --- | --- | --- | --- | --- | --- | --- | --- |
| Index N |  | 8 | 20 | 30 | 31 | 45 | 27 | 382 |
| Total | n | 8 | 20 | 30 | 31 | 45 | 27 | 382 |
|  | Mean (SD) | 82.12 (9.85) | 76.53 (8.14) | 87.74 (6.45) | 79.14 (13.73) | 81.06 (12.51) | 82.33 (9.35) | 80.73 (12.86) |
|  | Median | 79.17 | 75.83 | 88.69 | 82.61 | 81.52 | 81.52 | 82.61 |
|  | Min–Max | 71.53–97.92 | 63.33–97.78 | 73.81–100.00 | 42.39–98.91 | 46.74–100.00 | 64.13–98.86 | 25.00–100.00 |
| Physical Summary | n | 8 | 20 | 30 | 31 | 45 | 27 | 382 |
|  | Mean (SD) | 86.13 (10.31) | 82.17 (8.25) | 91.25 (8.89) | 84.88 (17.14) | 87.43 (14.80) | 89.35 (6.50) | 83.96 (17.55) |
|  | Median | 84.38 | 83.55 | 93.75 | 87.50 | 93.75 | 90.62 | 88.16 |
|  | Min–Max | 71.88–98.44 | 57.89–96.05 | 68.75–100.00 | 28.12–100.00 | 43.75–100.00 | 75.00–100.00 | 6.25–100.00 |
| Psychosocial Summary | n | 8 | 20 | 30 | 31 | 45 | 27 | 382 |
|  | Mean (SD) | 78.91 (12.56) | 72.17 (10.69) | 85.58 (8.46) | 76.08 (13.84) | 77.67 (14.51) | 78.58 (12.70) | 78.62 (13.57) |
|  | Median | 76.25 | 73.56 | 86.54 | 76.67 | 78.33 | 80.00 | 80.77 |
|  | Min–Max | 61.25–97.50 | 52.88–99.04 | 65.38–100.00 | 43.33–98.33 | 46.67–100.00 | 51.67–98.21 | 30.00–100.00 |
| Emotional | n | 8 | 20 | 30 | 31 | 45 | 27 | 382 |
|  | Mean (SD) | 73.96 (14.48) | 67.29 (15.41) | 76.00 (14.59) | 67.42 (15.54) | 72.89 (17.73) | 75.69 (19.15) | 73.61 (16.23) |
|  | Median | 69.79 | 68.75 | 75.00 | 65.00 | 75.00 | 75.00 | 75.00 |
|  | Min–Max | 60.42–95.83 | 43.75–97.92 | 45.00–100.00 | 35.00–100.00 | 40.00–100.00 | 15.00–100.00 | 15.00–100.00 |
| Social | n | 8 | 20 | 30 | 31 | 45 | 27 | 381 |
|  | Mean (SD) | 90.62 (10.02) | 89.44 (10.25) | 93.33 (7.35) | 84.84 (14.35) | 86.22 (15.89) | 82.96 (15.21) | 87.83 (14.83) |
|  | Median | 93.75 | 90.00 | 95.00 | 90.00 | 90.00 | 85.00 | 93.75 |
|  | Min–Max | 75.00–100.00 | 68.75–100.00 | 75.00–100.00 | 50.00–100.00 | 35.00–100.00 | 50.00–100.00 | 15.00–100.00 |
| Cognitive/School | n | 8 | 19 | 30 | 31 | 45 | 27 | 371 |
|  | Mean (SD) | 82.03 (15.10) | 69.30 (18.58) | 88.61 (10.83) | 75.97 (18.46) | 73.89 (18.80) | 77.04 (14.23) | 77.03 (18.61) |
|  | Median | 84.38 | 72.22 | 91.67 | 75.00 | 75.00 | 80.00 | 80.00 |
|  | Min–Max | 50.00–100.00 | 25.00–100.00 | 66.67–100.00 | 35.00–100.00 | 35.00–100.00 | 40.00–100.00 | 25.00–100.00 |

*Note. One global first eligible parent-proxy assessment per patient (N = 382); values were recalculated from raw items under the ≥50%-answered rule. n is the calculable-score denominator. All forms refers to all 382 patients across both recall versions. SD = standard deviation. Infant and Generic Core forms have different item sets; comparisons are descriptive.*

**Supplementary Table S1 (continued)**

*Panel C. Parent-proxy diagnostic-group rank comparisons*

| **Outcome** | **n acute / scar** | **Median acute** | **Median scar** | **U** | **Cliff’s δ** | **p** | **Holm p 7 tests** | **Holm p 6 scores** |
| --- | --- | --- | --- | --- | --- | --- | --- | --- |
| Total | 328/54 | 82.61 | 83.70 | 8736.0 | -0.014 | .874 | .874 | .874 |
| Physical Summary | 328/54 | 87.50 | 93.75 | 6748.5 | -0.238 | .005 | .030 | .030 |
| Psychosocial Summary | 328/54 | 80.77 | 79.17 | 9724.0 | 0.098 | .248 | .745 | .745 |
| Emotional | 328/54 | 75.00 | 70.00 | 9697.5 | 0.095 | .262 | .745 | .745 |
| Social | 327/54 | 95.00 | 90.00 | 9878.0 | 0.119 | .150 | .696 | .696 |
| Cognitive/School | 317/54 | 80.00 | 76.39 | 9632.0 | 0.125 | .139 | .696 | .696 |
| Physical | 328/54 | 84.38 | 93.75 | 6230.5 | -0.296 | < .001 | .003 | — |

*Panel D. Parent-proxy HC3-robust OLS sensitivity*

| **Outcome** | **n** | **Difference** | **SE** | **95% CI** | **p** | **Holm p 8 tests** | **Holm p 6 scores** |
| --- | --- | --- | --- | --- | --- | --- | --- |
| Total | 382 | 0.54 | 1.70 | -2.80 to 3.88 | .751 | 1.000 | .855 |
| Physical Summary | 382 | 6.57 | 1.83 | 2.99 to 10.14 | < .001 | .002 | .002 |
| Psychosocial Summary | 382 | -2.28 | 1.93 | -6.06 to 1.50 | .238 | 1.000 | .855 |
| Emotional | 382 | -2.94 | 2.47 | -7.78 to 1.89 | .233 | 1.000 | .855 |
| Social | 381 | -3.30 | 2.41 | -8.02 to 1.42 | .171 | 1.000 | .855 |
| Cognitive/School | 371 | -3.40 | 2.56 | -8.41 to 1.61 | .184 | 1.000 | .855 |
| Physical | 382 | 8.97 | 1.87 | 5.31 to 12.62 | < .001 | < .001 | — |

Note. Acute and scar denote diagnostic groups, not recall versions. Cliff’s δ is acute minus scar; the OLS contrast is scar minus acute. Intervals are pointwise. Physical functioning is the separate component, not the Physical Summary Score in Infant forms. The Physical Symptoms contrast was withheld because the scar-related group contained one observation; its unavailable OLS test was assigned p = 1 only for the original eight-test Holm calculation. The six-score sensitivity excludes the separate Physical Functioning component. — = not part of the six-score family.

**Supplementary Table S1 (continued)**

*Panel E. Parent-proxy diagnostic-supergroup omnibus comparisons*

| **Outcome** | **Included groups** | **n** | **Kruskal–Wallis H** | **p** |
| --- | --- | --- | --- | --- |
| Total | 8 | 380 | 6.542 | .478 |
| Physical Summary | 8 | 380 | 20.948 | .004 |
| Psychosocial Summary | 8 | 380 | 3.670 | .817 |
| Emotional | 8 | 380 | 5.524 | .596 |
| Social | 8 | 379 | 8.430 | .296 |
| Cognitive/School | 8 | 369 | 5.629 | .584 |
| Physical | 8 | 380 | 20.860 | .004 |
| Physical symptoms | 7 | 123 | 9.420 | .151 |

Note. Each included diagnostic supergroup had at least five calculable scores. Omnibus p values are unadjusted. Physical Symptoms is restricted to Infant forms; this omnibus test is distinct from the withheld two-group contrast.

*Panel F. Child self-report common-item known-groups sensitivity*

| **Outcome** | **n acute / scar** | **Median acute** | **Median scar** | **U** | **Cliff’s δ** | **p** | **Holm p** |
| --- | --- | --- | --- | --- | --- | --- | --- |
| Total | 90/43 | 84.09 | 85.23 | 1974.5 | 0.020 | .851 | 1.000 |
| Physical Summary | 90/43 | 87.50 | 90.62 | 1590.0 | -0.178 | .096 | .540 |
| Psychosocial Summary | 90/43 | 83.93 | 82.14 | 2247.5 | 0.161 | .133 | .540 |
| Emotional | 90/43 | 81.25 | 81.25 | 2231.5 | 0.153 | .151 | .540 |
| Social | 90/43 | 92.50 | 90.00 | 2013.5 | 0.041 | .700 | 1.000 |
| Cognitive/School | 90/43 | 82.50 | 75.00 | 2285.5 | 0.181 | .090 | .540 |

Note. Sc113 was excluded from all four child forms; original Emotional/Psychosocial/Total minimums of 3/8/12 were retained. Holm adjustment covers six outcomes. The reduced-item ≥50% sensitivity gave identical findings. Physical, Social, and Cognitive/School scores do not include Sc113.

**Supplementary Table S2**

*Estimation Diagnostics, Bootstrap Confidence Intervals and Caution-Flag Reasons for Every Omega Coefficient*

| **Form** | **Scale** | ***n*** | **Items** | **ω** | **95% CI** | **RMSR** | **Successful / requested bootstrap fits** | **Caution flags or exact reason for unavailable result** |
| --- | --- | --- | --- | --- | --- | --- | --- | --- |
| 1–12 mo. acute | Physical | 29 | 16 | .824* | — | .151 | 1305/2000 | n < 50; RMSR > .08; CI not reported: 1305/2000 successful (<90% success) |
|  | Emotional | 29 | 12 | .906* | [.826, .947] | .141 | 1982/2000 | n < 50; RMSR > .08 |
|  | Social | 29 | 4 | — | — | — | Not attempted | ω not estimated: improper/Heywood solution (non-positive residual variance) |
|  | Cognitive/School | 29 | 4 | .816* | — | .009 | 1536/2000 | n < 50; CI not reported: 1536/2000 successful (<90% success) |
|  | Psychosocial Summary | 29 | 20 | .918* | — | .164 | 898/2000 | n < 50; RMSR > .08; CI not reported: 898/2000 successful (<90% success) |
|  | Total | 29 | 36 | — | — | — | Not attempted | ω not estimated: complete-case n ≤ items (pragmatic protocol rule) |
| 13–24 mo. acute | Physical | 68 | 19 | .823* | [.720, .877] | .115 | 1925/2000 | RMSR > .08 |
|  | Emotional | 70 | 12 | .857* | [.800, .898] | .088 | 2000/2000 | RMSR > .08 |
|  | Social | 70 | 5 | .739* | [.605, .850] | .098 | 1970/2000 | RMSR > .08; CI width > .20 |
|  | Cognitive/School | 67 | 9 | .921* | [.881, .951] | .118 | 2000/2000 | RMSR > .08 |
|  | Psychosocial Summary | 65 | 26 | .935* | [.911, .953] | .098 | 1974/2000 | RMSR > .08 |
|  | Total | 63 | 45 | .945* | — | .110 | 1751/2000 | RMSR > .08; CI not reported: 1751/2000 successful (<90% success) |
| 2–4 y acute | Physical | 46 | 8 | .888* | [.344, .949] | .103 | 1973/2000 | n < 50; RMSR > .08; CI width > .20 |
|  | Emotional | 46 | 5 | .731* | [.546, .848] | .071 | 1958/2000 | n < 50; CI width > .20 |
|  | Social | 46 | 5 | .892* | [.604, .960] | .057 | 1843/2000 | n < 50; CI width > .20 |
|  | Cognitive/School | 37 | 3 | .742* | — | .000 | 948/2000 | n < 50; CI not reported: 948/2000 successful (<90% success) |
|  | Psychosocial Summary | 37 | 13 | .710* | — | .149 | 633/2000 | n < 50; RMSR > .08; CI not reported: 633/2000 successful (<90% success) |
|  | Total | 37 | 21 | .814* | — | .145 | 350/2000 | n < 50; RMSR > .08; CI not reported: 350/2000 successful (<500 valid fits and <90% success) |

**Supplementary Table S2 (continued)**

| **Form** | **Scale** | ***n*** | **Items** | **ω** | **95% CI** | **RMSR** | **Successful / requested bootstrap fits** | **Caution flags or exact reason for unavailable result** |
| --- | --- | --- | --- | --- | --- | --- | --- | --- |
| 5–7 y acute | Physical | 29 | 8 | .928* | [.854, .963] | .095 | 1996/2000 | n < 50; RMSR > .08 |
|  | Emotional | 29 | 5 | .763* | [.607, .874] | .123 | 1923/2000 | n < 50; RMSR > .08; CI width > .20 |
|  | Social | 29 | 5 | .836* | — | .143 | 1674/2000 | n < 50; RMSR > .08; CI not reported: 1674/2000 successful (<90% success) |
|  | Cognitive/School | 29 | 5 | — | — | — | Not attempted | ω not estimated: improper/Heywood solution (non-positive residual variance) |
|  | Psychosocial Summary | 29 | 15 | — | — | — | Not attempted | ω not estimated: optimizer boundary; unconstrained admissibility not established |
|  | Total | 29 | 23 | — | — | — | Not attempted | ω not estimated: optimizer boundary; unconstrained admissibility not established |
| 8–12 y acute parent | Physical | 41 | 8 | .934* | [.863, .967] | .079 | 2000/2000 | n < 50 |
|  | Emotional | 41 | 5 | .759* | [.639, .852] | .075 | 1883/2000 | n < 50; CI width > .20 |
|  | Social | 41 | 5 | .624* | [.297, .814] | .202 | 1821/2000 | n < 50; RMSR > .08; negative loading(s); CI width > .20 |
|  | Cognitive/School | 40 | 5 | .678* | — | .191 | 1742/2000 | n < 50; RMSR > .08; CI not reported: 1742/2000 successful (<90% success) |
|  | Psychosocial Summary | 40 | 15 | .855* | — | .172 | 1321/2000 | n < 50; RMSR > .08; CI not reported: 1321/2000 successful (<90% success) |
|  | Total | 40 | 23 | .914* | — | .193 | 834/2000 | n < 50; RMSR > .08; negative loading(s); CI not reported: 834/2000 successful (<90% success) |
| 8–12 y acute child | Physical | 39 | 8 | .916* | [.852, .953] | .065 | 1999/2000 | n < 50 |
|  | Emotional | 21 | 5 | .936* | [.875, .969] | .070 | 1950/2000 | n < 50 |
|  | Social | 39 | 5 | .857* | [.764, .924] | .143 | 1828/2000 | n < 50; RMSR > .08 |
|  | Cognitive/School | 39 | 5 | .881* | [.813, .932] | .104 | 1990/2000 | n < 50; RMSR > .08 |
|  | Psychosocial Summary | 21 | 15 | — | — | — | Not attempted | ω not estimated: improper/Heywood solution (non-positive residual variance) |
|  | Total | 21 | 23 | — | — | — | Not attempted | ω not estimated: complete-case n ≤ items (pragmatic protocol rule) |

**Supplementary Table S2 (continued)**

| **Form** | **Scale** | ***n*** | **Items** | **ω** | **95% CI** | **RMSR** | **Successful / requested bootstrap fits** | **Caution flags or exact reason for unavailable result** |
| --- | --- | --- | --- | --- | --- | --- | --- | --- |
| 13–18 y acute parent | Physical | 18 | 8 | .914* | — | .108 | 1604/2000 | n < 50; RMSR > .08; CI not reported: 1604/2000 successful (<90% success) |
|  | Emotional | 17 | 5 | .855* | — | .071 | 1767/2000 | n < 50; CI not reported: 1767/2000 successful (<90% success) |
|  | Social | 18 | 5 | .570* | — | .128 | 993/2000 | n < 50; RMSR > .08; negative loading(s); CI not reported: 993/2000 successful (<90% success) |
|  | Cognitive/School | 18 | 5 | .516* | — | .269 | 1694/2000 | n < 50; RMSR > .08; negative loading(s); CI not reported: 1694/2000 successful (<90% success) |
|  | Psychosocial Summary | 17 | 15 | .862* | — | .181 | 617/2000 | n < 50; RMSR > .08; negative loading(s); CI not reported: 617/2000 successful (<90% success) |
|  | Total | 17 | 23 | — | — | — | Not attempted | ω not estimated: complete-case n ≤ items (pragmatic protocol rule) |
| 13–18 y acute child | Physical | 18 | 8 | .926* | — | .122 | 1106/2000 | n < 50; RMSR > .08; CI not reported: 1106/2000 successful (<90% success) |
|  | Emotional | 13 | 5 | .869* | — | .057 | 1395/2000 | n < 50; CI not reported: 1395/2000 successful (<90% success) |
|  | Social | 18 | 5 | .501* | — | .290 | 1142/2000 | n < 50; RMSR > .08; CI not reported: 1142/2000 successful (<90% success) |
|  | Cognitive/School | 17 | 5 | .600* | [.006, .914] | .271 | 1800/2000 | n < 50; RMSR > .08; negative loading(s); CI width > .20 |
|  | Psychosocial Summary | 12 | 15 | — | — | — | Not attempted | ω not estimated: complete-case n ≤ items (pragmatic protocol rule) |
|  | Total | 12 | 23 | — | — | — | Not attempted | ω not estimated: complete-case n ≤ items (pragmatic protocol rule) |
| 1–12 mo. chronic | Physical | 13 | 16 | — | — | — | Not attempted | ω not estimated: complete-case n ≤ items (pragmatic protocol rule) |
|  | Emotional | 13 | 12 | .828* | — | .205 | 1637/2000 | n < 50; RMSR > .08; CI not reported: 1637/2000 successful (<90% success) |
|  | Social | 13 | 4 | — | — | — | Not attempted | ω not estimated: improper/Heywood solution (non-positive residual variance) |
|  | Cognitive/School | 13 | 4 | — | — | — | Not attempted | ω not estimated: improper/Heywood solution (non-positive residual variance) |
|  | Psychosocial Summary | 13 | 20 | — | — | — | Not attempted | ω not estimated: complete-case n ≤ items (pragmatic protocol rule) |
|  | Total | 13 | 36 | — | — | — | Not attempted | ω not estimated: complete-case n ≤ items (pragmatic protocol rule) |

**Supplementary Table S2 (continued)**

| **Form** | **Scale** | ***n*** | **Items** | **ω** | **95% CI** | **RMSR** | **Successful / requested bootstrap fits** | **Caution flags or exact reason for unavailable result** |
| --- | --- | --- | --- | --- | --- | --- | --- | --- |
| 13–24 mo. chronic | Physical | 46 | 19 | .875* | [.795, .920] | .132 | 1890/2000 | n < 50; RMSR > .08 |
|  | Emotional | 46 | 12 | .890* | [.840, .926] | .115 | 2000/2000 | n < 50; RMSR > .08 |
|  | Social | 45 | 5 | .837* | [.749, .908] | .077 | 1916/2000 | n < 50 |
|  | Cognitive/School | 45 | 9 | .847* | [.635, .921] | .176 | 2000/2000 | n < 50; RMSR > .08; CI width > .20 |
|  | Psychosocial Summary | 45 | 26 | .913* | [.850, .946] | .132 | 1946/2000 | n < 50; RMSR > .08 |
|  | Total | 45 | 45 | — | — | — | Not attempted | ω not estimated: complete-case n ≤ items (pragmatic protocol rule) |
| 2–4 y chronic | Physical | 50 | 8 | .904* | [.441, .968] | .099 | 1943/2000 | RMSR > .08; CI width > .20 |
|  | Emotional | 50 | 5 | .865 | [.744, .930] | .045 | 1894/2000 | — |
|  | Social | 50 | 5 | .815* | [.560, .926] | .146 | 1851/2000 | RMSR > .08; CI width > .20 |
|  | Cognitive/School | 50 | 3 | — | — | — | Not attempted | ω not estimated: improper/Heywood solution (non-positive residual variance) |
|  | Psychosocial Summary | 50 | 13 | — | — | — | Not attempted | ω not estimated: optimizer boundary; unconstrained admissibility not established |
|  | Total | 50 | 21 | — | — | — | Not attempted | ω not estimated: optimizer boundary; unconstrained admissibility not established |
| 5–7 y chronic | Physical | 44 | 8 | .881* | [.659, .944] | .146 | 1943/2000 | n < 50; RMSR > .08; CI width > .20 |
|  | Emotional | 44 | 5 | .836* | [.753, .892] | .046 | 1983/2000 | n < 50 |
|  | Social | 44 | 5 | .796* | — | .220 | 1768/2000 | n < 50; RMSR > .08; CI not reported: 1768/2000 successful (<90% success) |
|  | Cognitive/School | 44 | 5 | .809* | [.702, .881] | .162 | 1903/2000 | n < 50; RMSR > .08 |
|  | Psychosocial Summary | 44 | 15 | .916* | — | .134 | 1459/2000 | n < 50; RMSR > .08; CI not reported: 1459/2000 successful (<90% success) |
|  | Total | 44 | 23 | .938* | — | .165 | 897/2000 | n < 50; RMSR > .08; factor r > .95; CI not reported: 897/2000 successful (<90% success) |

**Supplementary Table S2 (continued)**

| **Form** | **Scale** | ***n*** | **Items** | **ω** | **95% CI** | **RMSR** | **Successful / requested bootstrap fits** | **Caution flags or exact reason for unavailable result** |
| --- | --- | --- | --- | --- | --- | --- | --- | --- |
| 8–12 y chronic parent | Physical | 69 | 8 | .841* | [.781, .891] | .111 | 1992/2000 | RMSR > .08 |
|  | Emotional | 69 | 5 | .809 | [.740, .866] | .048 | 1996/2000 | — |
|  | Social | 69 | 5 | .760* | [.519, .867] | .227 | 1939/2000 | RMSR > .08; CI width > .20 |
|  | Cognitive/School | 69 | 5 | .817* | [.734, .877] | .205 | 1998/2000 | RMSR > .08 |
|  | Psychosocial Summary | 69 | 15 | .904* | [.859, .934] | .126 | 1953/2000 | RMSR > .08 |
|  | Total | 69 | 23 | .920* | [.877, .945] | .145 | 1882/2000 | RMSR > .08 |
| 8–12 y chronic child | Physical | 67 | 8 | .761* | [.578, .861] | .118 | 1994/2000 | RMSR > .08; CI width > .20 |
|  | Emotional | 67 | 4 | .818* | [.731, .887] | .049 | 1994/2000 | Sc113 omitted |
|  | Social | 66 | 5 | .804* | [.652, .890] | .083 | 1953/2000 | RMSR > .08; CI width > .20 |
|  | Cognitive/School | 66 | 5 | .860* | [.767, .910] | .149 | 2000/2000 | RMSR > .08 |
|  | Psychosocial Summary | 65 | 14 | .927* | [.891, .952] | .089 | 1915/2000 | Sc113 omitted; RMSR > .08 |
|  | Total | 65 | 22 | .929* | — | .115 | 1349/2000 | Sc113 omitted; RMSR > .08; CI not reported: 1349/2000 successful (<90% success) |

**Supplementary Table S2 (continued)**

| **Form** | **Scale** | ***n*** | **Items** | **ω** | **95% CI** | **RMSR** | **Successful / requested bootstrap fits** | **Caution flags or exact reason for unavailable result** |
| --- | --- | --- | --- | --- | --- | --- | --- | --- |
| 13–18 y chronic parent | Physical | 36 | 8 | .497* | — | .156 | 1476/2000 | n < 50; RMSR > .08; CI not reported: 1476/2000 successful (<90% success) |
|  | Emotional | 35 | 5 | .863* | [.747, .930] | .079 | 1864/2000 | n < 50 |
|  | Social | 36 | 5 | .754* | — | .124 | 1504/2000 | n < 50; RMSR > .08; CI not reported: 1504/2000 successful (<90% success) |
|  | Cognitive/School | 36 | 5 | .587* | — | .236 | 1662/2000 | n < 50; RMSR > .08; CI not reported: 1662/2000 successful (<90% success) |
|  | Psychosocial Summary | 35 | 15 | — | — | — | Not attempted | ω not estimated: optimizer boundary; unconstrained admissibility not established |
|  | Total | 35 | 23 | — | — | — | Not attempted | ω not estimated: optimizer boundary; unconstrained admissibility not established |
| 13–18 y chronic child | Physical | 38 | 8 | .680* | — | .170 | 1799/2000 | n < 50; RMSR > .08; CI not reported: 1799/2000 successful (<90% success) |
|  | Emotional | 39 | 4 | .878* | — | .021 | 1481/2000 | Sc113 omitted; n < 50; CI not reported: 1481/2000 successful (<90% success) |
|  | Social | 38 | 5 | .830* | [.687, .909] | .108 | 1929/2000 | n < 50; RMSR > .08; CI width > .20 |
|  | Cognitive/School | 39 | 5 | .849* | — | .150 | 1707/2000 | n < 50; RMSR > .08; CI not reported: 1707/2000 successful (<90% success) |
|  | Psychosocial Summary | 38 | 14 | .930* | — | .139 | 994/2000 | Sc113 omitted; n < 50; RMSR > .08; CI not reported: 994/2000 successful (<90% success) |
|  | Total | 38 | 22 | .930* | — | .150 | 889/2000 | Sc113 omitted; n < 50; RMSR > .08; CI not reported: 889/2000 successful (<90% success) |

*Note.* Form codes and age groups are defined in Supplementary Table S3; acute = 7-day recall and chronic = 30-day recall. n is the scale-specific complete-case sample and Items the number of items analysed. Values are omega total for the equally weighted observed item score from prespecified correlated common-factor models fitted by unweighted minimum-residual least squares. *At least one descriptive caution flag applied. Percentile 95% confidence intervals were based on 2,000 requested patient-level bootstrap replications and reported only when at least 500 fits were valid and at least 90% of attempted fits succeeded. When omega was unavailable, bootstrapping was not attempted. Reasons distinguish the pragmatic sample-size rule (n ≤ items), an improper/Heywood solution, an optimizer boundary, and formal model non-identification; no coefficient was withheld for formal non-identification. RMSR is the root mean square off-diagonal residual of the reliability model. Flags are descriptive pragmatic thresholds rather than validated cutoffs. Sc113 was entirely unobserved and omitted in W6 and W8; affected coefficients use reduced item sets. In A6 and A8, Sc113 was unavailable before January 2024, so Emotional, Psychosocial Summary, and Total complete cases comprise later records only. An unavailable omega or confidence interval is not evidence of low reliability.

**Supplementary Table S3**

*Crosswalk of the 16 PedsQL™ Forms, Item Sets, and Score Construction*

| **Form** | **Age group** | **Recall** | **Items intended / available** | **Physical Summary Score (composition; minimum answered)** | **Psychosocial Summary Score (composition; minimum answered)** | **Total Score (composition; minimum answered)** |
| --- | --- | --- | --- | --- | --- | --- |
| ***Panel A: parent-proxy reports*** | | | | | | |
| A1 | 1–12 months | 7 days | 36 / 36 | PF (6) + PS (10) = 16; min. 8 | EF (12) + SF (4) + CF (4) = 20; min. 10 | All 36 items; min. 18 |
| W1 | 1–12 months | 30 days | 36 / 36 | PF (6) + PS (10) = 16; min. 8 | EF (12) + SF (4) + CF (4) = 20; min. 10 | All 36 items; min. 18 |
| A2 | 13–24 months | 7 days | 45 / 45 | PF (9) + PS (10) = 19; min. 10 | EF (12) + SF (5) + CF (9) = 26; min. 13 | All 45 items; min. 23 |
| W2 | 13–24 months | 30 days | 45 / 45 | PF (9) + PS (10) = 19; min. 10 | EF (12) + SF (5) + CF (9) = 26; min. 13 | All 45 items; min. 23 |
| A3 | 2–4 years | 7 days | 21 / 21 | PF (8); min. 4 | EF (5) + SF (5) + CF (3) = 13; min. 7 | All 21 items; min. 11 |
| W3 | 2–4 years | 30 days | 21 / 21 | PF (8); min. 4 | EF (5) + SF (5) + CF (3) = 13; min. 7 | All 21 items; min. 11 |
| A4 | 5–7 years | 7 days | 23 / 23 | PF (8); min. 4 | EF (5) + SF (5) + CF (5) = 15; min. 8 | All 23 items; min. 12 |
| W4 | 5–7 years | 30 days | 23 / 23 | PF (8); min. 4 | EF (5) + SF (5) + CF (5) = 15; min. 8 | All 23 items; min. 12 |
| A5 | 8–12 years | 7 days | 23 / 23 | PF (8); min. 4 | EF (5) + SF (5) + CF (5) = 15; min. 8 | All 23 items; min. 12 |
| W5 | 8–12 years | 30 days | 23 / 23 | PF (8); min. 4 | EF (5) + SF (5) + CF (5) = 15; min. 8 | All 23 items; min. 12 |
| A7 | 13–18 years | 7 days | 23 / 23 | PF (8); min. 4 | EF (5) + SF (5) + CF (5) = 15; min. 8 | All 23 items; min. 12 |
| W7 | 13–18 years | 30 days | 23 / 23 | PF (8); min. 4 | EF (5) + SF (5) + CF (5) = 15; min. 8 | All 23 items; min. 12 |
| ***Panel B: child self-reports*** | | | | | | |
| A6 | 8–12 years | 7 days | 23 / 23 | PF (8); min. 4 | EF (5) + SF (5) + CF (5) = 15; min. 8 | All 23 items; min. 12 |
| W6 | 8–12 years | 30 days | 23 / 22ᵃ | PF (8); min. 4 | EF (4) + SF (5) + CF (5) = 14; min. 8 | All 22 available items; min. 12 |
| A8 | 13–18 years | 7 days | 23 / 23 | PF (8); min. 4 | EF (5) + SF (5) + CF (5) = 15; min. 8 | All 23 items; min. 12 |
| W8 | 13–18 years | 30 days | 23 / 22ᵃ | PF (8); min. 4 | EF (4) + SF (5) + CF (5) = 14; min. 8 | All 22 available items; min. 12 |

*Note.* A = acute questionnaire (7-day recall); W = chronic questionnaire (30-day recall); PF = Physical Functioning; PS = Physical Symptoms; EF = Emotional Functioning; SF = Social Functioning; CF = Cognitive/School Functioning. ‘Min.’ denotes the minimum number of answered items required under the ≥50%-answered scoring rule. In the Infant Scales, the Physical Summary Score includes PF and PS; in the Generic Core Scales, it includes PF only. ᵃ In W6 and W8, EF item 3 (Sc113; ‘I feel angry’) was lost during transfer from the tablet system to the patient information system; the available EF, Psychosocial Summary, and Total scores therefore contain 4, 14, and 22 items, respectively. In A6 and A8, Sc113 responses from records completed before January 2024 were lost in the same transfer; affected scores were calculated from the available items under the same minimum requirements.

**Supplementary Table S4**

*Floor and Ceiling Effects by Questionnaire Form and PedsQL Scale*

| **Form** | **Scale** | **Calculable N** | **Floor n** | **Floor %** | **Ceiling n** | **Ceiling %** |
| --- | --- | --- | --- | --- | --- | --- |
| **Panel A: Parent-proxy reports** | | | | | | |
| A1 1–12 mo. acute parent proxy | Total | 30 | 0 | 0.0 | 1 | 3.3 |
|  | Physical Summary | 30 | 0 | 0.0 | 3 | 10.0 |
|  | Psychosocial Summary | 30 | 0 | 0.0 | 1 | 3.3 |
|  | Emotional | 30 | 0 | 0.0 | 3 | 10.0 |
|  | Social | 29 | 0 | 0.0 | 16 | 55.2 |
|  | Cognitive/School | 29 | 0 | 0.0 | 9 | 31.0 |
| A2 13–24 mo. acute parent proxy | Total | 72 | 0 | 0.0 | 2 | 2.8 |
|  | Physical Summary | 72 | 0 | 0.0 | 4 | 5.6 |
|  | Psychosocial Summary | 72 | 0 | 0.0 | 2 | 2.8 |
|  | Emotional | 72 | 0 | 0.0 | 2 | 2.8 |
|  | Social | 72 | 0 | 0.0 | 25 | 34.7 |
|  | Cognitive/School | 71 | 0 | 0.0 | 11 | 15.5 |
| A3 2–4 y acute parent proxy | Total | 47 | 0 | 0.0 | 2 | 4.3 |
|  | Physical Summary | 47 | 0 | 0.0 | 6 | 12.8 |
|  | Psychosocial Summary | 47 | 0 | 0.0 | 5 | 10.6 |
|  | Emotional | 47 | 0 | 0.0 | 5 | 10.6 |
|  | Social | 47 | 0 | 0.0 | 25 | 53.2 |
|  | Cognitive/School | 40 | 0 | 0.0 | 15 | 37.5 |
| A4 5–7 y acute parent proxy | Total | 29 | 0 | 0.0 | 3 | 10.3 |
|  | Physical Summary | 29 | 0 | 0.0 | 5 | 17.2 |
|  | Psychosocial Summary | 29 | 0 | 0.0 | 3 | 10.3 |
|  | Emotional | 29 | 0 | 0.0 | 5 | 17.2 |
|  | Social | 29 | 0 | 0.0 | 12 | 41.4 |
|  | Cognitive/School | 29 | 0 | 0.0 | 5 | 17.2 |

| **Form** | **Scale** | **Calculable N** | **Floor n** | **Floor %** | **Ceiling n** | **Ceiling %** |
| --- | --- | --- | --- | --- | --- | --- |
| **Panel A: Parent-proxy reports (continued)** | | | | | | |
| A5 8–12 y acute parent proxy | Total | 41 | 0 | 0.0 | 2 | 4.9 |
|  | Physical Summary | 41 | 0 | 0.0 | 8 | 19.5 |
|  | Psychosocial Summary | 41 | 0 | 0.0 | 2 | 4.9 |
|  | Emotional | 41 | 0 | 0.0 | 3 | 7.3 |
|  | Social | 41 | 0 | 0.0 | 12 | 29.3 |
|  | Cognitive/School | 40 | 0 | 0.0 | 6 | 15.0 |
| A7 13–18 y acute parent proxy | Total | 18 | 0 | 0.0 | 0 | 0.0 |
|  | Physical Summary | 18 | 0 | 0.0 | 3 | 16.7 |
|  | Psychosocial Summary | 18 | 0 | 0.0 | 0 | 0.0 |
|  | Emotional | 18 | 0 | 0.0 | 3 | 16.7 |
|  | Social | 18 | 0 | 0.0 | 8 | 44.4 |
|  | Cognitive/School | 18 | 0 | 0.0 | 1 | 5.6 |
| W1 1–12 mo. chronic parent proxy | Total | 13 | 0 | 0.0 | 0 | 0.0 |
|  | Physical Summary | 13 | 0 | 0.0 | 0 | 0.0 |
|  | Psychosocial Summary | 13 | 0 | 0.0 | 0 | 0.0 |
|  | Emotional | 13 | 0 | 0.0 | 0 | 0.0 |
|  | Social | 13 | 0 | 0.0 | 4 | 30.8 |
|  | Cognitive/School | 13 | 0 | 0.0 | 3 | 23.1 |
| W2 13–24 mo. chronic parent proxy | Total | 46 | 0 | 0.0 | 0 | 0.0 |
|  | Physical Summary | 46 | 0 | 0.0 | 1 | 2.2 |
|  | Psychosocial Summary | 46 | 0 | 0.0 | 0 | 0.0 |
|  | Emotional | 46 | 0 | 0.0 | 1 | 2.2 |
|  | Social | 46 | 0 | 0.0 | 21 | 45.7 |
|  | Cognitive/School | 45 | 0 | 0.0 | 9 | 20.0 |

| **Form** | **Scale** | **Calculable N** | **Floor n** | **Floor %** | **Ceiling n** | **Ceiling %** |
| --- | --- | --- | --- | --- | --- | --- |
| **Panel A: Parent-proxy reports (continued)** | | | | | | |
| W3 2–4 y chronic parent proxy | Total | 50 | 0 | 0.0 | 4 | 8.0 |
|  | Physical Summary | 50 | 1 | 2.0 | 16 | 32.0 |
|  | Psychosocial Summary | 50 | 0 | 0.0 | 6 | 12.0 |
|  | Emotional | 50 | 1 | 2.0 | 8 | 16.0 |
|  | Social | 50 | 0 | 0.0 | 23 | 46.0 |
|  | Cognitive/School | 50 | 0 | 0.0 | 21 | 42.0 |
| W4 5–7 y chronic parent proxy | Total | 44 | 0 | 0.0 | 1 | 2.3 |
|  | Physical Summary | 44 | 0 | 0.0 | 10 | 22.7 |
|  | Psychosocial Summary | 44 | 0 | 0.0 | 1 | 2.3 |
|  | Emotional | 44 | 0 | 0.0 | 2 | 4.5 |
|  | Social | 44 | 0 | 0.0 | 18 | 40.9 |
|  | Cognitive/School | 44 | 0 | 0.0 | 10 | 22.7 |
| W5 8–12 y chronic parent proxy | Total | 69 | 0 | 0.0 | 2 | 2.9 |
|  | Physical Summary | 69 | 0 | 0.0 | 17 | 24.6 |
|  | Psychosocial Summary | 69 | 0 | 0.0 | 2 | 2.9 |
|  | Emotional | 69 | 0 | 0.0 | 9 | 13.0 |
|  | Social | 69 | 0 | 0.0 | 22 | 31.9 |
|  | Cognitive/School | 69 | 0 | 0.0 | 9 | 13.0 |
| W7 13–18 y chronic parent proxy | Total | 36 | 0 | 0.0 | 1 | 2.8 |
|  | Physical Summary | 36 | 0 | 0.0 | 5 | 13.9 |
|  | Psychosocial Summary | 36 | 0 | 0.0 | 1 | 2.8 |
|  | Emotional | 36 | 0 | 0.0 | 6 | 16.7 |
|  | Social | 36 | 0 | 0.0 | 10 | 27.8 |
|  | Cognitive/School | 36 | 0 | 0.0 | 4 | 11.1 |

| **Form** | **Scale** | **Calculable N** | **Floor n** | **Floor %** | **Ceiling n** | **Ceiling %** |
| --- | --- | --- | --- | --- | --- | --- |
| **Panel B: Child self-reports** | | | | | | |
| A6 8–12 y acute child self-report | Total | 39 | 0 | 0.0 | 4 | 10.3 |
|  | Physical Summary | 39 | 0 | 0.0 | 6 | 15.4 |
|  | Psychosocial Summary | 39 | 0 | 0.0 | 6 | 15.4 |
|  | Emotional | 39 | 0 | 0.0 | 13 | 33.3 |
|  | Social | 39 | 0 | 0.0 | 15 | 38.5 |
|  | Cognitive/School | 39 | 0 | 0.0 | 11 | 28.2 |
| A8 13–18 y acute child self-report | Total | 18 | 0 | 0.0 | 0 | 0.0 |
|  | Physical Summary | 18 | 0 | 0.0 | 1 | 5.6 |
|  | Psychosocial Summary | 18 | 0 | 0.0 | 1 | 5.6 |
|  | Emotional | 18 | 0 | 0.0 | 5 | 27.8 |
|  | Social | 18 | 0 | 0.0 | 7 | 38.9 |
|  | Cognitive/School | 18 | 0 | 0.0 | 3 | 16.7 |
| W6 8–12 y chronic child self-report | Total | 67 | 0 | 0.0 | 5 | 7.5 |
|  | Physical Summary | 67 | 0 | 0.0 | 15 | 22.4 |
|  | Psychosocial Summary | 67 | 0 | 0.0 | 7 | 10.4 |
|  | Emotional | 67 | 0 | 0.0 | 14 | 20.9 |
|  | Social | 67 | 0 | 0.0 | 28 | 41.8 |
|  | Cognitive/School | 67 | 0 | 0.0 | 13 | 19.4 |
| W8 13–18 y chronic child self-report | Total | 39 | 0 | 0.0 | 4 | 10.3 |
|  | Physical Summary | 39 | 0 | 0.0 | 10 | 25.6 |
|  | Psychosocial Summary | 39 | 0 | 0.0 | 4 | 10.3 |
|  | Emotional | 39 | 0 | 0.0 | 9 | 23.1 |
|  | Social | 39 | 0 | 0.0 | 15 | 38.5 |
|  | Cognitive/School | 39 | 0 | 0.0 | 6 | 15.4 |

*Note.* N is the form- and scale-specific number of calculable scores; percentages are (n/N) × 100. Results use the first eligible assessment per patient within each form and the ≥50%-answered scoring rule. Floor = 0; ceiling = 100; percentages ≥15% indicate relevant effects. Acute = 7-day recall; chronic = 30-day recall, independently of longitudinal analysis windows. W6/W8 Emotional, Psychosocial Summary, and Total scores use reduced item sets because Sc113 was unavailable; the intended-form minimum numbers of answered items were retained. Earlier A6/A8 records without Sc113 were scored from the available items under the same rule.

**Supplementary Table S5**

*Paired Longitudinal Responsiveness, Assessment Intervals, and Questionnaire-Form Stability*

| **Outcome** | **Assessment interval, days** | | **Primary paired analysis** | | **Same recall** | | **Same age-specific form** | | **Same recall and form** | |
| --- | --- | --- | --- | --- | --- | --- | --- | --- | --- | --- |
|  | **Median (IQR)** | **Range** | **n** | **Mean change [95% CI]; adjusted p** | **n** | **Mean change [95% CI]; adjusted p** | **n** | **Mean change [95% CI]; adjusted p** | **n** | **Mean change [95% CI]; adjusted p** |
| Total Score | 119 (57–189) | 33–483 | 122 | 4.98 [2.80, 7.16]; < .001 | 69 | 3.54 [0.96, 6.11]; .039 | 98 | 5.15 [2.80, 7.49]; < .001 | 55 | 3.39 [0.84, 5.95]; .051 |
| Physical Summary | 119 (57–189) | 33–483 | 122 | 6.63 [3.45, 9.82]; < .001 | 69 | 5.27 [1.81, 8.73]; .020 | 98 | 6.45 [3.06, 9.84]; .001 | 55 | 4.99 [1.58, 8.41]; .029 |
| Psychosocial Summary | 119 (57–189) | 33–483 | 122 | 4.12 [1.93, 6.31]; .001 | 69 | 2.53 [-0.25, 5.30]; .220 | 98 | 4.52 [2.12, 6.93]; .001 | 55 | 2.51 [-0.50, 5.51]; .301 |
| Emotional functioning | 119 (57–189) | 33–483 | 122 | 4.31 [1.40, 7.22]; .009 | 69 | 1.56 [-2.24, 5.37]; .778 | 98 | 4.40 [1.10, 7.70]; .019 | 55 | 1.43 [-2.96, 5.82]; 1.000 |
| Social functioning | 119 (57–189) | 33–483 | 122 | 2.35 [0.00, 4.69]; .050 | 69 | 1.43 [-1.86, 4.73]; .778 | 98 | 2.28 [-0.20, 4.77]; .071 | 55 | 1.16 [-2.56, 4.88]; 1.000 |
| Cognitive/School functioning | 119 (56–189) | 33–483 | 121 | 5.34 [1.85, 8.83]; .009 | 69 | 4.43 [0.33, 8.53]; .138 | 97 | 7.21 [3.67, 10.75]; < .001 | 55 | 5.46 [1.12, 9.81]; .059 |
| **Questionnaire-form and recall stability for Total Score pairs** | | | | | | | | | | |
| **Stability category** | | | **n** | **% of 122** | | **Detail** | | | | |
| Same age-specific form | | | 98 | 80.3 | | Same parent-proxy age-form family at both selected assessments | | | | |
| Changed age-specific form | | | 24 | 19.7 | | Any change in the administered age-form family | | | | |
| Between Infant Scale age forms | | | 6 | 4.9 | | Five 1–12 to 13–24 months; one 13–24 to 1–12 months | | | | |
| Between Infant and Generic Core forms | | | 6 | 4.9 | | Five Infant-to-Generic; one Generic-to-Infant transition | | | | |
| Between Generic Core age forms | | | 12 | 9.8 | | Twelve transitions between Generic Core age-form families | | | | |
| Same questionnaire recall | | | 69 | 56.6 | | Acute-to-acute or chronic-to-chronic questionnaire recall | | | | |
| Same recall and same age-specific form | | | 55 | 45.1 | | Recall period and age-form item set both unchanged | | | | |
| Same recall but changed age-specific form | | | 14 | 11.5 | | Recall stability alone did not ensure item-set stability | | | | |
| Changed recall but same age-specific form | | | 43 | 35.2 | | Age-form item set stable despite recall change | | | | |

*Note.* Change is follow-up minus acute assessment-window score; positive values indicate higher HRQoL. Intervals describe the selected paired assessments. Confidence intervals are pointwise 95% intervals. Adjusted p values use the Holm procedure across the six outcomes separately within each analysis population. Same recall does not necessarily retain the same age-specific questionnaire; same age-specific form retains the parent-proxy item set but may change recall. Age-specific form denotes the administered form family, not independently verified chronological age. IQR = interquartile range.

**Supplementary Table S6**

*Linear Mixed-Effects Models of Longitudinal PedsQL™ Scores*

| **Outcome** | **Patients, n** | **Observations, n** | **Estimate** | **SE** | **Pointwise 95% CI** | **Raw p** | **Holm-adjusted p** | **Random-intercept variance** | **Residual variance** |
| --- | --- | --- | --- | --- | --- | --- | --- | --- | --- |
| **Panel A: Binary follow-up-window model** | | | | | | | | | |
| Total Score | 122 | 397 | 3.78 | 1.20 | [1.43, 6.12] | .002 | .008 | 62.55 | 104.63 |
| Physical Summary | 122 | 397 | 4.93 | 1.48 | [2.03, 7.82] | < .001 | .005 | 50.95 | 162.04 |
| Psychosocial Summary | 122 | 397 | 3.09 | 1.23 | [0.69, 5.49] | .012 | .039 | 92.89 | 108.76 |
| Emotional functioning | 122 | 397 | 3.88 | 1.50 | [0.93, 6.83] | .010 | .039 | 157.28 | 163.41 |
| Social functioning | 122 | 397 | 0.75 | 1.32 | [-1.85, 3.35] | .571 | .571 | 68.50 | 128.43 |
| Cognitive/School functioning | 121 | 394 | 3.80 | 1.68 | [0.50, 7.10] | .024 | .048 | 119.25 | 205.88 |
| **Panel B: Continuous-time model per 30 days** | | | | | | | | | |
| Total Score | 154 | 467 | 0.09 | 0.11 | [-0.12, 0.31] | .394 | 1.000 | 67.32 | 101.96 |
| Physical Summary | 154 | 467 | 0.30 | 0.14 | [0.03, 0.56] | .028 | .169 | 61.85 | 163.98 |
| Psychosocial Summary | 154 | 467 | -0.02 | 0.11 | [-0.24, 0.20] | .836 | 1.000 | 89.66 | 103.99 |
| Emotional functioning | 153 | 465 | 0.03 | 0.14 | [-0.24, 0.30] | .809 | 1.000 | 148.88 | 156.93 |
| Social functioning | 154 | 467 | -0.17 | 0.12 | [-0.40, 0.06] | .145 | .723 | 66.94 | 117.98 |
| Cognitive/School functioning | 152 | 462 | 0.01 | 0.16 | [-0.30, 0.32] | .953 | 1.000 | 111.03 | 218.78 |

*Note.* Models were estimated by maximum likelihood with a patient-specific random intercept and conditionally independent homoscedastic residuals, adjusted for questionnaire recall version, baseline age group, and sex. The binary model includes all calculable repeated observations from patients represented in both assessment windows; its estimate compares follow-up (≥31 days) with the acute window (0–30 days). The continuous model includes all calculable records from patients with at least two distinct score dates; its estimate is the change per 30 days. Confidence intervals and raw p values are model-based pointwise Wald results. Holm adjustment was applied across the six outcomes separately within each model type. All models converged without reportable warnings.

**Supplementary Table S6 (continued)**

*Cluster-Robust OLS Sensitivity Analyses*

| **Outcome** | **Patients, n** | **Observations, n** | **Estimate** | **SE** | **Pointwise 95% CI** | **Raw p** | **Holm-adjusted p** |
| --- | --- | --- | --- | --- | --- | --- | --- |
| **Panel C: Binary-window cluster-robust OLS** | | | | | | | |
| Total Score | 122 | 397 | 3.72 | 1.57 | [0.64, 6.79] | .018 | .078 |
| Physical Summary | 122 | 397 | 4.59 | 1.86 | [0.94, 8.23] | .014 | .078 |
| Psychosocial Summary | 122 | 397 | 3.32 | 1.56 | [0.26, 6.38] | .033 | .100 |
| Emotional functioning | 122 | 397 | 4.02 | 1.91 | [0.27, 7.77] | .036 | .100 |
| Social functioning | 122 | 397 | 0.68 | 1.76 | [-2.77, 4.13] | .699 | .699 |
| Cognitive/School functioning | 121 | 394 | 4.48 | 1.80 | [0.94, 8.01] | .013 | .078 |
| **Panel D: Continuous-time cluster-robust OLS per 30 days** | | | | | | | |
| Total Score | 154 | 467 | -0.06 | 0.22 | [-0.50, 0.37] | .773 | 1.000 |
| Physical Summary | 154 | 467 | 0.14 | 0.24 | [-0.33, 0.62] | .553 | 1.000 |
| Psychosocial Summary | 154 | 467 | -0.17 | 0.22 | [-0.60, 0.26] | .444 | 1.000 |
| Emotional functioning | 153 | 465 | -0.29 | 0.22 | [-0.72, 0.14] | .186 | 1.000 |
| Social functioning | 154 | 467 | -0.22 | 0.26 | [-0.74, 0.29] | .396 | 1.000 |
| Cognitive/School functioning | 152 | 462 | 0.02 | 0.19 | [-0.34, 0.39] | .895 | 1.000 |

*Note.* OLS models used the same outcomes, analytical samples, fixed-effect covariates, and time terms as Panels A and B. Standard errors used a patient-clustered sandwich covariance estimator with the default finite-sample correction in statsmodels. Confidence intervals and raw p values are pointwise normal-Wald results; Holm adjustment was applied across six outcomes separately for each time specification. OLS = ordinary least squares; SE = standard error.

**Supplementary Table S7**

*Confirmatory Factor Analysis Sensitivity Fit and Standardized Parameters*

**Panel A. Robust maximum-likelihood sensitivity model fit**

| **Analysis** | **Model** | **n** | **χ²** | **df** | **p** | **CFI** | **TLI** | **RMSEA [90% CI]** | **SRMR** | **AIC** | **BIC** |
| --- | --- | --- | --- | --- | --- | --- | --- | --- | --- | --- | --- |
| Primary 21-item | Four-factor correlated | 244 | 611.42 | 183 | < .001 | .777 | .744 | .108 [.098, .117] | .133 | 12345.6 | 12586.9 |
| Primary 21-item | Two-factor | 244 | 759.84 | 188 | < .001 | .698 | .663 | .124 [.114, .133] | .136 | 12526.1 | 12749.9 |
| Primary 21-item | Second-order | 244 | 617.64 | 185 | < .001 | .774 | .744 | .108 [.098, .117] | .134 | 12349.5 | 12583.8 |
| Sensitivity 23-item | Four-factor correlated | 179 | 768.91 | 224 | < .001 | .699 | .660 | .127 [.117, .137] | .165 | 10396.7 | 10635.8 |
| Sensitivity 23-item | Two-factor | 179 | 901.07 | 229 | < .001 | .628 | .589 | .139 [.129, .149] | .161 | 10544.1 | 10767.2 |
| Sensitivity 23-item | Second-order | 179 | 775.70 | 226 | < .001 | .696 | .659 | .127 [.117, .137] | .167 | 10400.4 | 10633.1 |

Note. Panel A reports MLR sensitivity fits. Robust CFI, TLI, and RMSEA are shown; RMSEA intervals are 90% intervals. SRMR is the conventional lavaan SRMR. All samples were complete cases; MLR used a mean structure and missing = "fiml". CFI = comparative fit index; TLI = Tucker–Lewis index; RMSEA = root mean square error of approximation; SRMR = standardized root mean square residual; AIC/BIC = Akaike/Bayesian information criterion.

**Supplementary Table S7 (continued)**

*Panel B. Standardized item loadings — Primary 21-item*

| **Intended domain** | **Item** | **WLSMV 4F** | **WLSMV 2F** | **WLSMV HO** | **MLR 4F** | **MLR 2F** | **MLR HO** |
| --- | --- | --- | --- | --- | --- | --- | --- |
| Physical | Sc011 | 0.904 | 0.904 | 0.904 | 0.846 | 0.844 | 0.844 |
| Physical | Sc012 | 0.982 | 0.982 | 0.982 | 0.893 | 0.893 | 0.894 |
| Physical | Sc013 | 0.878 | 0.878 | 0.878 | 0.863 | 0.865 | 0.865 |
| Physical | Sc014 | 0.858 | 0.858 | 0.859 | 0.760 | 0.760 | 0.759 |
| Physical | Sc015 | 0.786 | 0.785 | 0.786 | 0.696 | 0.695 | 0.695 |
| Physical | Sc016 | 0.781 | 0.783 | 0.783 | 0.598 | 0.598 | 0.597 |
| Physical | Sc017 | 0.568 | 0.565 | 0.565 | 0.437 | 0.436 | 0.436 |
| Physical | Sc018 | 0.621 | 0.621 | 0.620 | 0.364 | 0.362 | 0.361 |
| Emotional | Sc111 | 0.731 | 0.630 | 0.728 | 0.644 | 0.488 | 0.638 |
| Emotional | Sc112 | 0.696 | 0.599 | 0.699 | 0.683 | 0.496 | 0.684 |
| Emotional | Sc113 | 0.673 | 0.587 | 0.675 | 0.621 | 0.556 | 0.639 |
| Emotional | Sc114 | 0.652 | 0.570 | 0.647 | 0.531 | 0.486 | 0.527 |
| Emotional | Sc115 | 0.709 | 0.617 | 0.713 | 0.652 | 0.544 | 0.643 |
| Social | Sc211 | 0.835 | 0.760 | 0.837 | 0.855 | 0.695 | 0.847 |
| Social | Sc212 | 0.687 | 0.633 | 0.689 | 0.714 | 0.563 | 0.708 |
| Social | Sc213 | 0.725 | 0.685 | 0.727 | 0.640 | 0.593 | 0.635 |
| Social | Sc214 | 0.794 | 0.708 | 0.787 | 0.196 | 0.290 | 0.227 |
| Social | Sc215 | 0.802 | 0.743 | 0.801 | 0.495 | 0.487 | 0.517 |
| Cognitive/School | Sc311 | 0.834 | 0.698 | 0.838 | 0.748 | 0.612 | 0.746 |
| Cognitive/School | Sc312 | 0.736 | 0.605 | 0.729 | 0.694 | 0.572 | 0.694 |
| Cognitive/School | Sc313 | 0.828 | 0.697 | 0.830 | 0.746 | 0.611 | 0.749 |

Note. Fully standardized loadings (std.all). 4F = four correlated domains; 2F = correlated Physical and Psychosocial factors; HO = second-order HRQoL model. In the 2F model, Emotional, Social, and Cognitive/School items load on Psychosocial. WLSMV uses ordered 0–4 items and theta parameterization; MLR treats items as continuous. Both use std.lv = TRUE. Primary n = 244; sensitivity n = 179. Item codes and factor assignments match the executable syntax.

**Supplementary Table S7 (continued)**

*Panel C. Standardized item loadings — Sensitivity 23-item*

| **Intended domain** | **Item** | **WLSMV 4F** | **WLSMV 2F** | **WLSMV HO** | **MLR 4F** | **MLR 2F** | **MLR HO** |
| --- | --- | --- | --- | --- | --- | --- | --- |
| Physical | Sc011 | 0.904 | 0.906 | 0.905 | 0.848 | 0.846 | 0.847 |
| Physical | Sc012 | 0.983 | 0.982 | 0.983 | 0.895 | 0.896 | 0.897 |
| Physical | Sc013 | 0.901 | 0.902 | 0.901 | 0.882 | 0.884 | 0.885 |
| Physical | Sc014 | 0.869 | 0.869 | 0.869 | 0.761 | 0.762 | 0.761 |
| Physical | Sc015 | 0.830 | 0.829 | 0.829 | 0.749 | 0.747 | 0.747 |
| Physical | Sc016 | 0.793 | 0.794 | 0.794 | 0.610 | 0.609 | 0.607 |
| Physical | Sc017 | 0.615 | 0.612 | 0.614 | 0.514 | 0.512 | 0.512 |
| Physical | Sc018 | 0.658 | 0.660 | 0.659 | 0.396 | 0.393 | 0.392 |
| Emotional | Sc111 | 0.734 | 0.637 | 0.734 | 0.661 | 0.505 | 0.656 |
| Emotional | Sc112 | 0.689 | 0.595 | 0.691 | 0.665 | 0.492 | 0.667 |
| Emotional | Sc113 | 0.691 | 0.601 | 0.689 | 0.629 | 0.599 | 0.649 |
| Emotional | Sc114 | 0.752 | 0.655 | 0.750 | 0.613 | 0.592 | 0.603 |
| Emotional | Sc115 | 0.713 | 0.620 | 0.714 | 0.680 | 0.549 | 0.672 |
| Social | Sc211 | 0.817 | 0.725 | 0.818 | 0.881 | 0.683 | 0.874 |
| Social | Sc212 | 0.676 | 0.606 | 0.678 | 0.722 | 0.553 | 0.719 |
| Social | Sc213 | 0.669 | 0.620 | 0.672 | 0.628 | 0.569 | 0.623 |
| Social | Sc214 | 0.871 | 0.763 | 0.867 | 0.154 | 0.287 | 0.186 |
| Social | Sc215 | 0.776 | 0.707 | 0.775 | 0.474 | 0.464 | 0.495 |
| Cognitive/School | Sc311 | 0.789 | 0.687 | 0.789 | 0.799 | 0.630 | 0.798 |
| Cognitive/School | Sc312 | 0.632 | 0.551 | 0.628 | 0.666 | 0.587 | 0.664 |
| Cognitive/School | Sc313 | 0.883 | 0.757 | 0.880 | 0.870 | 0.722 | 0.870 |
| Cognitive/School | Sc314 | 0.734 | 0.632 | 0.739 | 0.102 | 0.234 | 0.105 |
| Cognitive/School | Sc315 | 0.748 | 0.637 | 0.750 | 0.131 | 0.292 | 0.134 |

Note. Fully standardized loadings (std.all). 4F = four correlated domains; 2F = correlated Physical and Psychosocial factors; HO = second-order HRQoL model. In the 2F model, Emotional, Social, and Cognitive/School items load on Psychosocial. WLSMV uses ordered 0–4 items and theta parameterization; MLR treats items as continuous. Both use std.lv = TRUE. Primary n = 244; sensitivity n = 179. Item codes and factor assignments match the executable syntax.

**Supplementary Table S7 (continued)**

*Panel D. Fully standardized higher-order domain loadings*

| **Domain** | **21 items WLSMV** | **21 items MLR** | **23 items WLSMV** | **23 items MLR** |
| --- | --- | --- | --- | --- |
| Physical | 0.531 | 0.200 | 0.600 | 0.169 |
| Emotional | 0.771 | 0.727 | 0.796 | 0.757 |
| Social | 0.871 | 0.783 | 0.865 | 0.774 |
| Cognitive/School | 0.692 | 0.769 | 0.692 | 0.769 |

*Panel E. Latent correlations — Primary 21-item*

| **Factor pair** | **WLSMV 4F** | **WLSMV 2F** | **WLSMV HO** | **MLR 4F** | **MLR 2F** | **MLR HO** |
| --- | --- | --- | --- | --- | --- | --- |
| Physical / Emotional | 0.447 | — | 0.410 | 0.269 | — | 0.145 |
| Physical / Social | 0.489 | — | 0.463 | 0.062 | — | 0.156 |
| Physical / Cognitive/School | 0.258 | — | 0.368 | 0.148 | — | 0.154 |
| Emotional / Social | 0.612 | — | 0.671 | 0.564 | — | 0.570 |
| Emotional / Cognitive/School | 0.583 | — | 0.534 | 0.537 | — | 0.559 |
| Social / Cognitive/School | 0.644 | — | 0.603 | 0.614 | — | 0.602 |
| Physical / Psychosocial | — | 0.476 | — | — | 0.220 | — |

*Panel F. Latent correlations — Sensitivity 23-item*

| **Factor pair** | **WLSMV 4F** | **WLSMV 2F** | **WLSMV HO** | **MLR 4F** | **MLR 2F** | **MLR HO** |
| --- | --- | --- | --- | --- | --- | --- |
| Physical / Emotional | 0.479 | — | 0.478 | 0.268 | — | 0.128 |
| Physical / Social | 0.545 | — | 0.519 | 0.028 | — | 0.131 |
| Physical / Cognitive/School | 0.377 | — | 0.415 | 0.113 | — | 0.130 |
| Emotional / Social | 0.653 | — | 0.688 | 0.577 | — | 0.586 |
| Emotional / Cognitive/School | 0.596 | — | 0.551 | 0.566 | — | 0.583 |
| Social / Cognitive/School | 0.600 | — | 0.598 | 0.606 | — | 0.595 |
| Physical / Psychosocial | — | 0.536 | — | — | 0.219 | — |

Note. HO correlations among first-order domains are model-implied through the higher-order factor, not freely estimated domain covariances. Correlations of each domain with HRQoL equal the fully standardized higher-order loadings in Panel D. — = no corresponding parameter in that model. The warning and admissibility diagnostics are reported in Table S8; adequate item loadings do not imply adequate global fit.

**Supplementary Table S8**

*Numerical Diagnostics for the Confirmatory Factor Analysis Models*

| **Analysis** | **Estimator** | **Model** | **n** | **df** | **Max \|gradient\|** | **Minimum vcov eigenvalue** | **vcov rank/dimension** | **Minimum information eigenvalue** | **Maximum \|latent r\|** | **Admissible** |
| --- | --- | --- | --- | --- | --- | --- | --- | --- | --- | --- |
| Primary 21-item | WLSMV | Four-factor correlated | 244 | 183 | 2.40e-06 | -1.14e-16 | 107/108 | 6.16e-05 | 0.644 | Yes |
| Primary 21-item | WLSMV | Two-factor | 244 | 188 | 2.45e-06 | -2.49e-17 | 102/103 | 6.42e-05 | 0.476 | Yes |
| Primary 21-item | WLSMV | Second-order | 244 | 185 | 3.48e-06 | 4.23e-17 | 105/106 | 6.30e-05 | 0.871 | Yes |
| Primary 21-item | MLR | Four-factor correlated | 244 | 183 | 4.24e-06 | 7.57e-05 | 69/69 | 1.82e-01 | 0.614 | Yes |
| Primary 21-item | MLR | Two-factor | 244 | 188 | 1.79e-06 | 8.03e-05 | 64/64 | 1.80e-01 | 0.220 | Yes |
| Primary 21-item | MLR | Second-order | 244 | 185 | 4.31e-06 | 6.74e-05 | 67/67 | 4.79e-02 | 0.783 | Yes |
| Sensitivity 23-item | WLSMV | Four-factor correlated | 179 | 224 | 4.51e-06 | 3.34e-16 | 117/118 | 9.23e-05 | 0.653 | Yes |
| Sensitivity 23-item | WLSMV | Two-factor | 179 | 229 | 4.13e-06 | 1.50e-16 | 112/113 | 1.06e-04 | 0.536 | Yes |
| Sensitivity 23-item | WLSMV | Second-order | 179 | 226 | 4.61e-06 | 5.52e-17 | 115/116 | 1.04e-04 | 0.865 | Yes |
| Sensitivity 23-item | MLR | Four-factor correlated | 179 | 224 | 5.58e-06 | 7.94e-05 | 75/75 | 1.55e-01 | 0.606 | Yes |
| Sensitivity 23-item | MLR | Two-factor | 179 | 229 | 2.47e-06 | 8.51e-05 | 70/70 | 1.51e-01 | 0.219 | Yes |
| Sensitivity 23-item | MLR | Second-order | 179 | 226 | 7.05e-06 | 6.71e-05 | 73/73 | 6.28e-02 | 0.774 | Yes |

*Note.* All models converged and passed the lavaan post-estimation check. The numerical rank used a tolerance of 10⁻¹⁰ times the largest absolute eigenvalue. Each WLSMV parameter variance–covariance matrix contained one eigenvalue at numerical-zero magnitude; all expected-information matrices were full rank. No model showed a negative observed residual variance, negative latent variance, latent correlation with |r| ≥ 1, nonfinite standardized parameter, or standardized loading > 1. The corresponding MLR models had full-rank parameter variance–covariance matrices and generated no estimation warning. Thus, the WLSMV warning is most consistent with numerical near-singularity of the parameter covariance matrix rather than an improper solution, although standard errors and parameter-level inference remain subject to caution. vcov = variance–covariance matrix of the estimated parameters; information = expected information matrix.

**Supplementary Table S9**

*Exploratory Factor and Parallel-Analysis Results*

**Panel A. Factor-retention and final EFA diagnostics**

| **Analysis** | **Correlation** | **n** | **Items** | **Factors retained** | **EFA factors** | **RMSR** | **Minimum uniqueness** | **Diagnostic** |
| --- | --- | --- | --- | --- | --- | --- | --- | --- |
| Primary 21-item | Polychoric | 244 | 21 | 4 | 4 | 0.030 | 0.153 | Smoothed matrix; positive final uniquenesses |
|  | Pearson sensitivity | 244 | 21 | 4 | 4 | 0.026 | 0.257 | Positive final uniquenesses |
| Sensitivity 23-item | Polychoric | 179 | 23 | 5 | 5 | 0.027 | 0.043 | Smoothed matrix; ultra-Heywood warning |
|  | Pearson sensitivity | 179 | 23 | 4 | 4 | 0.032 | 0.264 | Positive final uniquenesses |

**Panel B. Observed and simulated factor eigenvalues**

| **Analysis** | **Factor** | **Polychoric observed** | **Polychoric 95th percentile** | **Polychoric retained** | **Pearson observed** | **Pearson 95th percentile** | **Pearson retained** |
| --- | --- | --- | --- | --- | --- | --- | --- |
| Primary 21-item | 1 | 6.939 | 0.792 | Yes | 5.430 | 0.808 | Yes |
| Primary 21-item | 2 | 3.637 | 0.563 | Yes | 3.010 | 0.551 | Yes |
| Primary 21-item | 3 | 0.964 | 0.467 | Yes | 0.852 | 0.466 | Yes |
| Primary 21-item | 4 | 0.549 | 0.396 | Yes | 0.464 | 0.393 | Yes |
| Primary 21-item | 5 | 0.117 | 0.337 | No | 0.096 | 0.330 | No |
| Primary 21-item | 6 | 0.084 | 0.277 | No | 0.091 | 0.276 | No |
| Sensitivity 23-item | 1 | 7.730 | 0.961 | Yes | 6.175 | 0.951 | Yes |
| Sensitivity 23-item | 2 | 4.003 | 0.702 | Yes | 3.391 | 0.699 | Yes |
| Sensitivity 23-item | 3 | 1.061 | 0.595 | Yes | 1.029 | 0.599 | Yes |
| Sensitivity 23-item | 4 | 0.631 | 0.503 | Yes | 0.566 | 0.510 | Yes |
| Sensitivity 23-item | 5 | 0.449 | 0.430 | Yes | 0.341 | 0.432 | No |
| Sensitivity 23-item | 6 | 0.128 | 0.365 | No | 0.148 | 0.363 | No |

*Note.* Analyses used the same complete-case parent-proxy samples as the CFA (21-item primary analysis: n = 244; 23-item sensitivity analysis: n = 179). Factor retention compared MINRES factor eigenvalues with their 95th percentiles across 1,000 independent random-normal datasets; factors were retained sequentially until the first observed eigenvalue failed to exceed its reference value. Exploratory models used MINRES extraction and oblimin rotation. Polychoric correlations were estimated without a continuity correction; both matrices required smoothing because they were not positive definite. The polychoric 23-item analysis retained a fifth factor only narrowly (0.449 vs 0.430) and its five-factor extraction generated an ultra-Heywood warning; this factor was largely defined by Sc314 and Sc315. Pearson sensitivity analyses retained four factors in both samples and yielded positive uniquenesses. These exploratory results were not used to modify the prespecified CFA models. EFA = exploratory factor analysis; MINRES = minimum residual; RMSR = root mean square residual.

**Supplementary Table S9 (continued)**

*Panel C. Polychoric EFA pattern loadings — Primary 21-item*

| **Item** | **MR1** | **MR4** | **MR2** | **MR3** | **Communality** | **Uniqueness** |
| --- | --- | --- | --- | --- | --- | --- |
| Sc011 | 0.894 | -0.045 | 0.099 | -0.145 | 0.828 | 0.172 |
| Sc012 | 0.920 | 0.026 | -0.011 | -0.006 | 0.847 | 0.153 |
| Sc013 | 0.925 | 0.012 | -0.049 | -0.043 | 0.836 | 0.164 |
| Sc014 | 0.823 | 0.162 | 0.038 | -0.007 | 0.751 | 0.249 |
| Sc015 | 0.807 | -0.024 | 0.102 | -0.048 | 0.683 | 0.317 |
| Sc016 | 0.635 | 0.224 | 0.163 | 0.035 | 0.597 | 0.403 |
| Sc017 | 0.439 | -0.178 | 0.399 | -0.020 | 0.386 | 0.614 |
| Sc018 | 0.355 | 0.018 | 0.475 | -0.087 | 0.407 | 0.593 |
| Sc111 | 0.172 | 0.094 | 0.624 | 0.013 | 0.526 | 0.474 |
| Sc112 | -0.014 | -0.073 | 0.784 | 0.134 | 0.642 | 0.358 |
| Sc113 | -0.211 | 0.315 | 0.533 | 0.073 | 0.535 | 0.465 |
| Sc114 | 0.163 | 0.368 | 0.396 | -0.089 | 0.421 | 0.579 |
| Sc115 | 0.093 | -0.010 | 0.537 | 0.283 | 0.484 | 0.516 |
| Sc211 | -0.118 | 0.179 | 0.171 | 0.761 | 0.842 | 0.158 |
| Sc212 | -0.183 | 0.129 | 0.125 | 0.680 | 0.627 | 0.373 |
| Sc213 | -0.176 | 0.334 | 0.161 | 0.499 | 0.611 | 0.389 |
| Sc214 | 0.775 | -0.016 | -0.135 | 0.321 | 0.698 | 0.302 |
| Sc215 | 0.508 | -0.030 | -0.007 | 0.684 | 0.772 | 0.228 |
| Sc311 | 0.129 | 0.766 | -0.118 | 0.156 | 0.693 | 0.307 |
| Sc312 | -0.042 | 0.774 | 0.017 | 0.001 | 0.604 | 0.396 |
| Sc313 | 0.086 | 0.774 | 0.065 | -0.022 | 0.648 | 0.352 |

Factor correlations: MR1/MR4 = 0.110; MR1/MR2 = 0.212; MR4/MR2 = 0.367; MR1/MR3 = 0.102; MR4/MR3 = 0.453; MR2/MR3 = 0.284.

Note. MINRES extraction with oblimin rotation after smoothing the polychoric matrix. MR labels preserve the returned software factor identifiers; they are not confirmatory domain labels. Values are rotated pattern coefficients, not structure coefficients. n = 244.

**Supplementary Table S9 (continued)**

*Panel D. Polychoric EFA pattern loadings — Sensitivity 23-item*

| **Item** | **MR1** | **MR4** | **MR2** | **MR3** | **MR5** | **Communality** | **Uniqueness** |
| --- | --- | --- | --- | --- | --- | --- | --- |
| Sc011 | -0.181 | 0.116 | -0.050 | 0.827 | 0.135 | 0.862 | 0.138 |
| Sc012 | 0.013 | 0.009 | 0.017 | 0.947 | -0.066 | 0.856 | 0.144 |
| Sc013 | -0.032 | -0.067 | -0.029 | 0.924 | 0.020 | 0.844 | 0.156 |
| Sc014 | 0.026 | -0.040 | 0.198 | 0.816 | 0.089 | 0.789 | 0.211 |
| Sc015 | -0.115 | 0.114 | 0.002 | 0.824 | 0.038 | 0.761 | 0.239 |
| Sc016 | -0.021 | 0.200 | 0.244 | 0.647 | 0.013 | 0.637 | 0.363 |
| Sc017 | -0.007 | 0.369 | -0.222 | 0.480 | 0.060 | 0.460 | 0.540 |
| Sc018 | -0.089 | 0.428 | 0.004 | 0.315 | 0.179 | 0.449 | 0.551 |
| Sc111 | 0.039 | 0.641 | 0.040 | 0.110 | 0.089 | 0.548 | 0.452 |
| Sc112 | 0.099 | 0.811 | -0.078 | 0.012 | -0.017 | 0.669 | 0.331 |
| Sc113 | 0.095 | 0.574 | 0.353 | -0.150 | -0.112 | 0.617 | 0.383 |
| Sc114 | -0.063 | 0.345 | 0.443 | 0.179 | 0.133 | 0.536 | 0.464 |
| Sc115 | 0.191 | 0.550 | 0.002 | 0.023 | 0.175 | 0.517 | 0.483 |
| Sc211 | 0.805 | 0.154 | 0.159 | -0.073 | 0.004 | 0.901 | 0.099 |
| Sc212 | 0.668 | 0.139 | 0.143 | -0.166 | 0.027 | 0.651 | 0.349 |
| Sc213 | 0.438 | 0.138 | 0.344 | -0.228 | 0.129 | 0.580 | 0.420 |
| Sc214 | 0.240 | -0.096 | -0.003 | 0.827 | 0.025 | 0.734 | 0.266 |
| Sc215 | 0.650 | -0.011 | -0.056 | 0.534 | 0.069 | 0.743 | 0.257 |
| Sc311 | 0.029 | -0.051 | 0.878 | 0.109 | -0.081 | 0.774 | 0.226 |
| Sc312 | 0.047 | 0.006 | 0.728 | -0.064 | 0.053 | 0.571 | 0.429 |
| Sc313 | 0.058 | 0.019 | 0.844 | 0.044 | 0.068 | 0.802 | 0.198 |
| Sc314 | -0.069 | -0.006 | 0.021 | -0.022 | 0.995 | 0.957 | 0.043 |
| Sc315 | 0.164 | -0.019 | -0.044 | 0.032 | 0.765 | 0.649 | 0.351 |

Factor correlations: MR3/MR2 = 0.077; MR3/MR4 = 0.226; MR2/MR4 = 0.349; MR3/MR1 = 0.041; MR2/MR1 = 0.443; MR4/MR1 = 0.311; MR3/MR5 = 0.419; MR2/MR5 = 0.110; MR4/MR5 = 0.314; MR1/MR5 = 0.148.

Note. MINRES extraction with oblimin rotation after smoothing the polychoric matrix. MR labels preserve the returned software factor identifiers; they are not confirmatory domain labels. Values are rotated pattern coefficients, not structure coefficients. n = 179. The 23-item extraction issued an ultra-Heywood warning although final reported uniquenesses were positive; interpret the exploratory five-factor solution cautiously.
