# Supplementary material for: Validity and reliability of the pediatric quality of life inventory™ 4.0 generic core scales to measure health-related quality of life in pediatric outpatients after thermal injuries
Source: J Patient Rep Outcomes. 2026 Nov 11;10:210. doi: 10.1186/s41687-026-01247-2 (PMC13759929; doi:10.1186/s41687-026-01247-2)
Supplement: Supplementary file 3 [file 41687_2026_1247_MOESM3_ESM.docx]

**Plain English summary**

Burns can affect children long after the wounds have healed, including their physical abilities, feelings, friendships and schooling. Clinics therefore need questionnaires that capture these aspects reliably, but no burn-specific questionnaire tested in German was available for children. We examined whether the German version of a widely used children's quality-of-life questionnaire, the PedsQL™, works in routine follow-up after burns. At one German children's burn clinic, parents and children completed 951 questionnaires between 2022 and 2026. About four in five planned questionnaires were completed; language barriers were the most common reason when they were not. Where this could be assessed, answers within each area of life were mostly consistent, and scores improved over time, most clearly for physical well-being. Parents reported slightly poorer physical well-being for children with recent burns than for children with scars. However, the expected structure of the questionnaire was only partly confirmed; many children had the best possible scores for social and school functioning, and some age versions were completed too rarely for firm conclusions. The questionnaire can help clinics monitor groups of children after burns alongside clinical assessment, but it should not be used on its own for decisions about individual children.
